# Patient-reported outcomes associated with cancer screening: a systematic review

**DOI:** 10.1186/s12885-022-09261-5

**Published:** 2022-03-01

**Authors:** Ashley Kim, Karen C. Chung, Christopher Keir, Donald L. Patrick

**Affiliations:** 1 GRAIL, LLC, a subsidiary of Illumina, Inc., CA Menlo Park, USA; 2 University of Washington, Seattle, WA USA

**Keywords:** Patient-reported outcomes, Patient-reported outcome measures, Clinical trials, Cancer screening

## Abstract

**Background:**

Multi-cancer early detection tests have been developed to enable earlier detection of multiple cancer types through screening. As reflected by patient-reported outcomes (PROs), the psychosocial impact of cancer screening is not yet clear. Our aim is to evaluate the impact of cancer screening through PRO assessment.

**Methods:**

A systematic review was conducted using MEDLINE, EMBASE, and reference lists of articles from January 2000 to August 2020 for relevant publications assessing the psychosocial impact of cancer screening before and within 1 year after screening in the general asymptomatic population, including following receipt of results. Studies focused on diagnostic evaluation or involving patients previously diagnosed with cancer were excluded.

**Results:**

In total, 31 studies (12 randomized controlled trials; 19 observational studies) were included, reflecting PRO assessments associated with lung, breast, colorectal, anal, ovarian, cervical, and prostate cancer screening procedures. The most commonly assessed construct was symptoms of anxiety, using the State-Trait Anxiety Inventory. Cancer-specific distress and worry were also assessed using a broad range of measures. Overall, individuals tolerated screening procedures well with no major psychosocial effects. Of note, increases in symptoms of anxiety and levels of distress and worry were generally found prior to communication of screening results and following communication of indeterminate or positive results that required further testing. These negative psychosocial effects were, however, not long-lasting and returned to baseline relatively soon after screening. Furthermore, individuals with higher cancer risk, such as current smokers and those with a family history of cancer, tended to have higher levels of anxiety and distress throughout the screening process, including following negative or indeterminate results.

**Conclusions:**

The psychosocial impact of cancer screening is relatively low overall and short-lived, even following false-positive test results. Individuals with a higher risk of cancer tend to experience more symptoms of anxiety and distress during the screening process; thus, more attention to this group is recommended.

**Supplementary Information:**

The online version contains supplementary material available at 10.1186/s12885-022-09261-5.

## Background

Cancer stage at diagnosis is an important prognostic indicator for patient outcomes, with detection at later stages predictive of mortality. Alongside nearly 1,806,590 new cancer cases and 606,520 cancer deaths projected to occur in the United States (US) in 2020 [1], there is a high incidence of later-stage cancer [2]. For instance, more than 50% of lung, colorectal, cervical, ovarian, and pancreatic cancers, 30% of breast cancers, and 20% of prostate cancers are diagnosed at later stages [3–5], when treatments are generally less effective. This demonstrates the need for continued progress in the area of early detection of cancer across cancer types through the use of cancer screening, which has been associated with decreased cancer morbidity and mortality [6–8]. Currently, novel cancer screening tests are being developed and implemented, including single-cancer screening tests such as breath biopsies for breast and lung cancer [9, 10], Cytosponge for esophageal cancer [11], and human papillomavirus testing for cervical cancer [12] as well as multi-cancer early detection approaches, which leverage advances such as machine learning [13–16], methylation detection [13, 17], or genome-wide fragmentation patterns [14].

The World Health Organization has defined the value of cancer screening as the ability to identify unrecognized (pre-clinical) cancer or pre-cancerous lesions in an apparently healthy target population [18]. The US Preventive Services Task Force (USPSTF) currently recommends age-specific, single-cancer screening for breast, colon, cervical, and lung cancer, with prostate cancer screening recommended as an individual decision, in select adults and patient populations at a higher risk of developing cancer [19–24]. Additionally, many experts also recommend patient population–specific screening for individuals with risk factors for anal, esophageal, gastric, and hepatobiliary cancers [25–28].

The USPSTF-recommended single-cancer screening modalities range from the more invasive colonoscopy and pap smear, to noninvasive imaging modalities such as mammography and low-dose computed tomography (LDCT), and to the blood-based prostate-specific antigen (PSA) test [20–24]. Of note, newer multi-cancer early detection tests, currently being developed in the form of blood-based tests, would enable earlier detection of multiple cancer types simultaneously [13]. As with any screening tool, it is important to consider both the benefits and harms of cancer screening. Although the benefits of screening and early detection are well recognized (e.g., finding cancers earlier when easier to treat, improved survival), the harms of screening potentially include overdiagnosis and overtreatment [19], false-positive results that may lead to additional testing and biopsies, and complications from additional testing. Additional impacts that are not yet well understood or well defined include the psychological and social aspects of screening, which may be quantified through the use of patient-reported outcome measures (PROMs), which consist of self-reported questionnaires that provide quantitative measures of a patient’s health condition directly from the patient [29]. PROMs evaluate specific constructs that comprise patient-reported outcomes (PROs), which are selected through qualitative research with patients and providers, as well as from the published literature. The continued proliferation of newer cancer screening tests makes the need to understand psychosocial outcomes even more important.

To date, based on existing qualitative research, negative screening results can be psychologically beneficial by virtue of the reassurance they provide [30] and can have a minimal impact on distress [31] and anxiety [32], respectively, whereas abnormal and false-positive screening results can have a negative impact on the following psychosocial concept domains: anxiety, fear, mood, behavior, sleep, sexuality, and social functioning, which includes stigmatization and relationships within one’s social network [33]. Literature on the psychosocial effects of cancer screening modalities in the general asymptomatic population, and the psychological sequelae of different screening test results in this population, is heterogeneous and has not been systematically reviewed and published. As a result, a comprehensive assessment of PROs or PROMs will not only capture both the positive and negative psychosocial consequences of cancer screening during the screening process, but also inform the value of effective communication and education strategies. Patient-reported outcomes can help screening be more widely understood, accepted, and recommended.

The objective of this systematic review is to evaluate the evidence regarding the psychosocial effects of cancer screening as measured by PROMs in the general asymptomatic population without cancer-related signs or symptoms, recognized disease, or prior screening. Here, we focus on the magnitude and duration of the psychosocial impact of the cancer screening process: prior to a screening to 1 year post-test, including return of test results (e.g., normal, abnormal, indeterminate). We identify relevant concepts assessed in cancer screening studies and their impact in different populations, including those at a higher risk of developing cancer. This review will help guide and further inform the implementation of PROMs in future clinical trials for cancer screening tests.

## Methods

### Search strategy

**Fig. 1:**
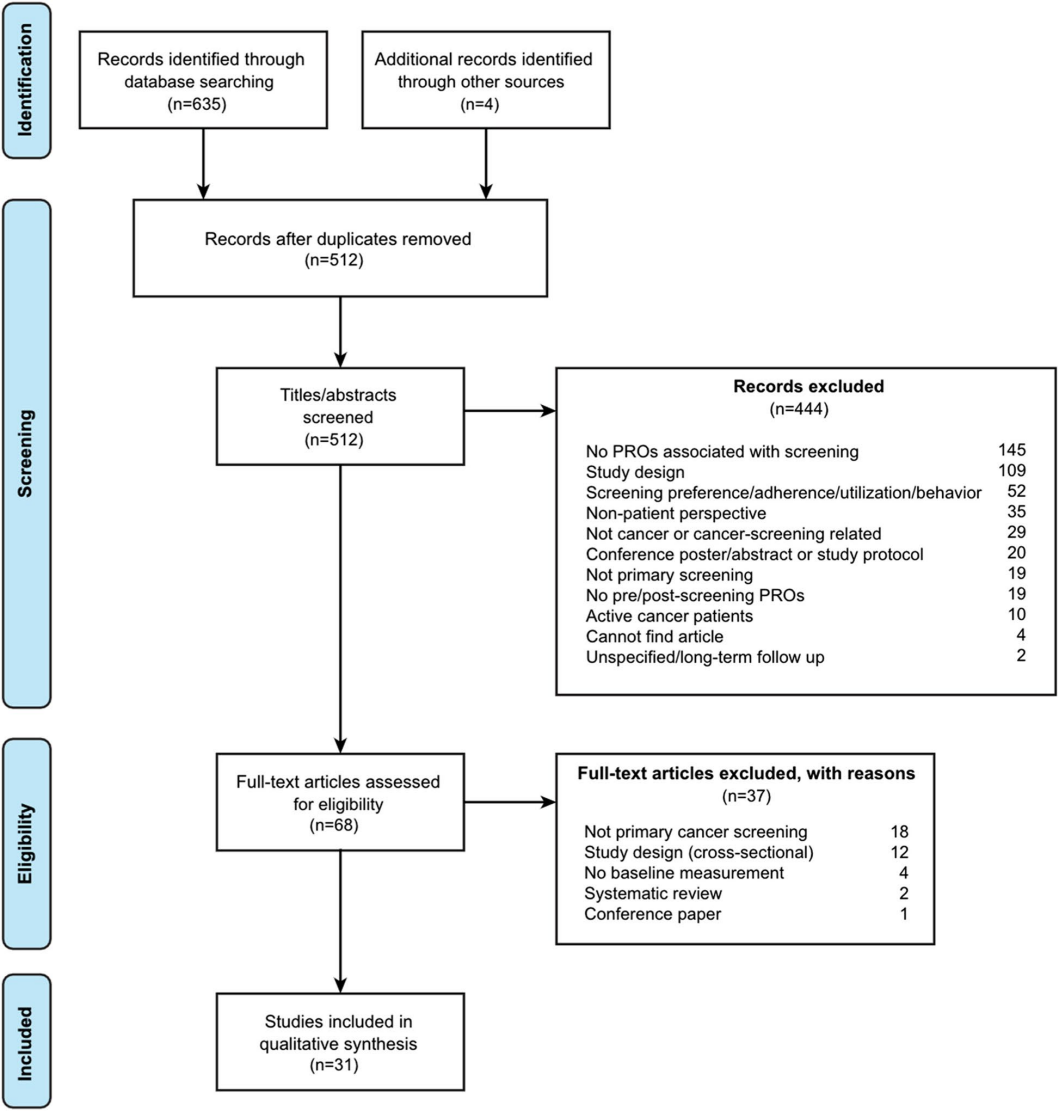
Preferred Reporting Items for Systematic Reviews and Meta-Analyses (PRISMA) Flow Diagram

We followed the Preferred Reporting Items for Systematic Reviews and Meta-Analyses (PRISMA) guidelines for this review, as shown in Fig. 1 and Additional file 1: Table S1. A systematic literature review was conducted using MEDLINE and EMBASE between January 2000 and August 2020, using a combination of keywords for cancer (e.g., neoplasms), cancer screening (e.g., early detection of cancer), and symptoms (e.g., anxiety, distress, worry), with terms for PROMs (e.g., questionnaire, surveys, PROs) combined with Boolean logic (and/or). The full search terms are available in Additional file 2: Tables S2, Additional file 3: Table S3. Reference lists from the articles returned from the searches were manually scanned for articles not identified through electronic means.

### Study selection

An initial screening of titles/abstracts was performed, followed by a full-text review. Studies were considered for inclusion if they included cancer screening in asymptomatic individuals (i.e., no signs, symptoms, or diagnoses), aged ≥18 years from a screening setting (e.g., screening program or clinical trial) inside or outside of the US, assessed the psychosocial impact of cancer screening via PROMs at ≥2 time points including pre- and post-screening (up to 1 year), included self-reported PROMs, reported screening results (e.g., false-positive outcomes), and were published in a peer-reviewed journal.

Manuscripts were excluded if they included patients previously or currently diagnosed with cancer or previously screened individuals with abnormal findings, assessed a diagnostic evaluation, included PROMs completed by proxy (e.g., clinician, caregiver, expert), or included PROMs assessing pharmacological (e.g., bowel preparation, on-demand sedation) or non-pharmacological (e.g., music therapy, mammography with implant) treatments or interventions associated with screening, or provider-specific screening (e.g., nurse practitioner, female doctor). Non-longitudinal (e.g., cross-sectional) studies or reviews were also excluded. Any discrepancies in study inclusion or exclusion were resolved among three reviewers (AK, KC, DLP).

### Data extraction

Studies that met the inclusion criteria were assessed. One author (AK) recorded the following details of each included study (Additional file 4: Table S4). A second author (KC or DLP) reviewed all abstractions for verification, completeness, and accuracy. Any discrepancies were discussed among the three reviewers until a consensus was reached.

### Quality of reporting

Following the PRISMA statement, a quality assessment of study design, data collection techniques, and analysis and interpretation of results was performed by three authors (AK, KC, DLP) using elements of critical appraisal from the checklists in the USPSTF Quality Rating Criteria for Randomized Controlled Trials (RCTs) and the National Institutes of Health (NIH) Quality Assessment Table for Observational Cohort Studies (Additional files 5: Tables S5, Additional file 6: Table S6). Any discrepancies in the risk of bias assessments were resolved among the three reviewers.

## Results

We identified 639 articles and excluded 444 at the title and abstract level after removing 195 duplicate records. After reviewing 68 full-text articles, 31 met study inclusion criteria. Study characteristics and findings from the 31 articles are included in the final full text review in Additional file 4: Table S4. Of the 31 articles, 19 were observational studies and 12 were RCTs, with two separate articles based on the Bowel Cancer Screening in Norway (BCSN) screening trial, and four separate articles based on the Dutch-Belgian Lung Cancer Screening (NELSON) trial.

We conducted a risk assessment of each study to check for quality by using two risk-of-bias checklists, given the two types of studies included in our review. In general, the quality of the RCTs included in our review was categorized as “good,” and most of the observational studies sufficiently defined the population, inclusion criteria, outcome measures, and the timeframe. In nearly half of the observational studies, sources of bias and confounding were addressed rigorously. Quality ratings are reported in Additional files 5: Tables S5, Additional file 6: Table S6.

We identified seven constructs that are assessed in cancer screening studies: symptoms of anxiety, symptoms of depression, distress, worry, functional status and well-being, preference-weighted health status, and other psychosocial. Our review focused on outcomes related to the first four constructs (Tables 1, 2, 3 and 4), while the remaining constructs can be found in Additional file 7: Tables S7. Additional file 8: Table S8, Additional file 9: Table S9.

**Table 1:** Patient-Reported Outcomes Related to Symptoms of Anxiety

	Study Design	Screening	Measure^†^	Result	Screening		2 mo	3 mo	4 mo	5 mo	6 mo	7 mo–11 mo	12 mo
					Baseline	Within 1 mo							
					Mean (SD)	Mean (SD)	Mean (SD)						
Taghizadeh et al 2019 [34] *N* = 953–1237 (Total) *N* = 238–279 (+) • Age: 50–75 years • Pan-Canadian Early Detection of Lung Cancer Study	Prospective cohort	LDCT	STAI-state (Form Y-1)	Total (+/−)	30.9	33.1*^,a^							31.7*^,a^
				(+)	29.9	33.2^a^							31.7^a^
Kirkoen et al 2016 [35] *N* = 1523–3462 • Age: 50–74 years • BCSN pilot participants	Randomized screening	FS or FIT	HADS-A	(+)	≈3.8^b^	≈4.0^b^					≈3.8^b^		≈3.7^b^
				(−)	≈3.6^b^	≈3.4^b^					≈3.6^b^		≈3.7^b^
Kirkoen et al 2016 [36] *N* = 1730–3521 • Age: 50–74 years • BCSN pilot participants	Randomized screening	FS	HADS-A	(+)	3.3 (2.4)	3.5 (2.7)							
				(−)	3.3 (2.4)	3.2 (2.4)*							
		FIT		(+)	3.1 (2.5)	3.3 (2.6)							
				(−)	3.6 (2.4)	3.4 (2.4)*							
Brain et al 2016 [37] *N* = 1579–2019 *N* = 73 (−) *N* = 41 (Incidental) *N* = 788 (+)/Repeat *N* = 48 (+)/MDT • Age: 50–75 years • UKLS trial participants	RCT	LDCT	HADS-A	Total (+/−/Incidental)	3.7	3.7 (3.5–3.8)							
				(−)		3.5 (3.4–3.7)							
				Incidental		3.5 (2.8–4.4)							
				(+)/Repeat scan		3.8 (3.6–4.0)							
				(+)/MDT referral		5.5 (4.5–6.7)*							
				Control	3.7	3.8 (3.6–3.9)							
Laing et al 2014 [38] *N* = 34–96 • Age: 50–74 years • No FOBT in last 8 months, no FS in last 4 years, no colonoscopy in last 9 years	Prospective longitudinal cohort	FOBT	STAI-state	(+)	35.6	38.8			34.6				
				(−)	32.5	30.9			30.6				
Korfage et al 2012 [17] *N* = 706–789 • Age: 30–60 years • Women living in Netherlands	Prospective cohort questionnaire	Pap	STAI-state (STAI-6)	Normal	33 (10)	32 (10)^c^	31 (10)*^,d^						
Hafslund et al 2012 [39] *N* = 77–128 • Age: 50–69 years	Prospective descriptive longitudinal	MMG	HADS-A	FP	3.9 (3.1)	4.6 (3.7)*^,e^		4.3 (3.4)			4.1 (3.2)		
				(−)	4.1 (3.3)	NR		NR			4.0 (3.3)		
Aggestrup et al 2012 [40] *N* = 1853–2052 • DLCST participants	RCT	LDCT	COS-LC	Normal	1.5 (2.2)	1.5 (2.5)^d^							
				Control	1.6 (2.3)	1.7 (2.8)^d^							
van den Bergh et al 2010 [41] *N* = 600–641 • Age: 50–75 years • NELSON study participants	RCT	LDCT	STAI-state (STAI-6)	Indeterminate	33.6 (9.3)	33.5 (8.9)^f^	34.8 (9.2)*						
				(−)	33.1 (8.4)	32.5 (8.8)^f^	32.6 (9.2)						
Wood et al 2008 [42] N = 15 • Age: ≥25 years • Referred women from HNPCC or Lynch syndrome families	Observational	OPH + EB + TVS + CA125	HADS-A	Total (−/FP)	7.7 (4.7)			7.6 (5.1)			7.3 (5.5)		
van den Bergh et al 2008 [43] *N* = 288–324 • Age: 50–75 years • NELSON study participants	RCT	LDCT	STAI-state (STAI-6)	Total (−/Incidental)	33.3^g^	30.0^g^					33.3^g^		
Byrne et al 2008 [44] *N* = 341 • Age: 50–79 years	Prospective cohort	LDCT	STAI-state	Suspicious	32.6 (12.3)	38.3 (14.4)					32.6 (12.1)		35.1 (17.5)
				Indeterminate	34.4 (12.3)	37.7 (13.8)*					37.3 (12.6)		35.3 (13.5)
				(−)	35.9 (12.4)	35.9 (12.3)					34.4 (12.0)		35.1 (12.9)
			STAI-trait	Suspicious	33.9 (9.8)	36.6 (11.2)					35.4 (11.7)		35.0 (16.3)
				Indeterminate	36.7 (11.7)	37.5 (12.2)					36.7 (11.9)		36.3 (12.4)
				(−)	37.0 (11.3)	36.6 (11.3)					35.7 (12.0)		35.8 (11.8)
Absetz et al 2003 [45] *N* = 141–621 • Age: 50–59 years • Some participants with familial history of breast cancer	Prospective questionnaire	MMG	STAI-state	High PS	37.2 (12.2)		37.2 (11.4)						35.9 (10.2)
				Mod PS	33.7 (9.2)		33.9 (9.2)						34.1 (9.9)
				Low PS	31.5 (9)		31.7 (8.4)						31.3 (10.1)
Cormier et al 2002 [46] *N* = 220 • Age: 40–70 years • Family history of prostate cancer	Prospective questionnaire	PSA	STAI-state	Normal	79 (17)^h^	79 (18)^c,h,i^	81 (17)^d,h,i^						

*Indicating statistical significance, *p* < 0.05

^†^STAI, HADS, DASS, COS: higher scores indicate more symptoms of anxiety

^a^Compared to baseline, more participants had a clinically significant increase (i.e., MCID ≥10) in symptoms of anxiety at 1 month following receipt of positive results, and to a lesser degree at 12 months

^b^Estimated numbers from figure in manuscript

^c^Post-screen, with no specific time point

^d^After receiving screen results, with no specific time point

^e^18% had a clinically significant level of anxiety at 2 weeks

^f^Post screen, before results

^g^Median values

^h^All scores are standardized from 0 (poorest health) to 100 (best health) within primary manuscript to simplify interpretation of results

^i^Fewer individuals had a clinically significant difference in symptoms of anxiety after receiving their results, compared to while waiting for their results

*Abbreviations*: *BCSN* Bowel Cancer Screening in Norway, *CA-125* ovarian tumor marker, *COS-LC* Consequences Of Screening in Lung Cancer, *DLCST* Danish Lung Cancer Screening Trial, *EB* endometrial biopsy, *FIT* fecal immunochemical test, *FP* false positive, *FOBT* fecal occult blood test, *FS* flexible sigmoidoscopy, *HADS-A* Hospital Anxiety and Depression Scale – Anxiety subscale, *HNPCC* hereditary nonpolyposis colorectal cancer, *LDCT* low-dose computed tomography, *MDT* multidisciplinary team, *MMG* mammogram, *Mod* moderate, *NELSON* The Dutch-Belgian Randomized Lung Cancer Screening Trial, *NR* not reported, *OPH* outpatient hysteroscopy, *PS* perceived susceptibility, *PSA* prostate-specific antigen, *RCT* randomized controlled trial, *SD* standard deviation, *STAI* State-Trait Anxiety Inventory, *STAI-A* State-Trait Anxiety Inventory – Anxiety subscale, *TVS* transvaginal ultrasound scan, *UKLS* United Kingdom Lung Screening

**Table 2:** Patient-Reported Outcomes Related to Symptoms of Depression

	Study Design	Screening	Measure^†^	Result	Screening		2 mo	3 mo	4 mo	5 mo	6 mo	7 mo–11 mo	12 mo
					Baseline	Within 1 mo							
					Mean (SD)	Mean (SD)	Mean (SD)						
Brain et al 2016 [37] N = 1579–2019 N = 73 (−) N = 41 (Incidental) N = 788 (+)/Repeat N = 48 (+)/MDT • Age: 50–75 years • UKLS trial participants	RCT	LDCT	HADS-D	Total (+/−/Incidental)	2.7	2.5 (2.4–2.6)							
				(−)		2.6 (2.4–2.7)							
				Incidental		2.3 (1.8–3.0)							
				(+)/Repeat scan		2.5 (2.4–2.7)							
				(+)/MDT referral		3.1 (2.4–3.8)							
				Control^a^	2.6	2.8 (2.7–2.9)*							
Kirkoen et al 2016 [35] N = 1523–3462 • Age: 50–74 years BCSN pilot participants	Randomized screening	FS or FIT	HADS-D	(+)	≈2.7^b^	NR^c^					NR		≈2.6^b^
				(−)	≈2.3^b^	NR^c^					NR		≈2.6^b^
Kirkoen et al 2016 [36] N = 1730–3521 • Age: 50–74 years • BCSN pilot participants	Randomized screening	FS	HADS-D	(+)	2.6 (2.1)	2.3 (2.3)^c^							
				(−)	2.4 (2.0)	2.4 (2.4)^c^							
		FIT		(+)	3.0 (2.1)	2.7 (2.2)^c^							
				(−)	2.5 (2.1)	2.5 (2.1)^c^							
Hafslund et al 2012 [39] N = 77–246 • Age: 50–69 years	Prospective descriptive longitudinal	MMG	HADS-D	FP	2.6 (2.2)	2.9 (3.0)		3.1 (2.9)			3.2 (2.9)*^,d^		
				(−)	2.4 (2.6)	NR		NR			2.4 (2.6)		
Wood et al 2008 [42] N = 15 • Age: ≥25 years • Referred women from HNPCC or Lynch syndrome families	Observational	OPH + EB + TVS + CA-125	HADS-D	Total (−/FP)	3.6 (5.1)			3.6 (5.4)			2.7 (3.2)		
Absetz et al 2003 [45] *N* = 145–643 • Age: 50–59 years • Some participants with familial history of breast cancer	Prospective questionnaire	MMG	BDI-I	High PS	9.2 (8.0)		8.5 (8.2)						9.3 (8.3)
				Mod PS	7.6 (7.3)		6.5 (7.0)						7.3 (7.8)
				Low PS	5.7 (6.3)		5.5 (6.5)						5.9 (7.7)

*Indicating statistical significance, *p* < 0.05, compared to the (−) group

^†^HADS, DASS, BDI: higher scores indicate more symptoms of depression

^a^Control group = no intervention

^b^Estimated numbers from figure in manuscript

^c^After receiving screen results, with no specific time point

^d^Indicating statistical significance, *p* < 0.05, compared to Control (−)

*Abbreviations*: *BCSN* Bowel Cancer Screening in Norway, *BDI* Beck’s Depression Inventory, *CA-125* ovarian tumor marker, *EB* endometrial biopsy, *FIT* fecal immunochemical test, *FS* flexible sigmoidoscopy, *FP* false positive, *HADS-D* Hospital Anxiety and Depression Scale – Depression subscale, *HNPCC* hereditary nonpolyposis colorectal cancer, *LDCT* low-dose computed tomography, *MDT* multidisciplinary team, *MMG* mammogram, *Mod* moderate, *NR* not reported, *OPH* outpatient hysteroscopy, *PS* perceived susceptibility, *RCT* randomized controlled trial, *SD* standard deviation, *TVS* transvaginal ultrasound scan, *UKLS* United Kingdom Lung Screening

**Table 3:** Patient-Reported Outcomes Related to Distress

	Study Design	Screening	Measure^†^	Result	Screening		2 mo	3 mo	4 mo	5 mo	6 mo	7 mo–11 mo	12 mo
					Baseline	Within 1 mo							
					Mean (SD)	Mean (SD)	Mean (SD)						
Ruberg et al 2016 [47] *N* = 180 (Total) *N* = 16 (Abnormal) • Age: 30–60 years • Women living in Netherlands	Prospective longitudinal	TVS/CA125	IES-intrusive	Total (Abnormal, Normal)	3.5 (5.6)	3.1 (5.0)	2.4 (4.8)						
				Abnormal	2.9 (4.4)	3.7 (4.7)	6.3 (6.4)						
				Normal	3.6 (5.7)	3.0 (5.1)	2.1 (4.4)*^,a^						
van den Bergh et al 2011 [48] N = 600–658 • Age: 50–75 years • NELSON study participants	RCT	LDCT	IES (total)	Indeterminate	4.0 (2.8–5.3)		7.8 (6.5–9.0)*^b^						
				(−)	4.1 (3.4–4.8)		2.6 (2.0–3.3)						
			IES-intrusive	Indeterminate	1.9 (1.3–2.4)		3.4 (2.8–3.9)						
				(−)	1.7 (1.4–2.0)		1.0 (0.7–1.3)						
			IES-avoidance	Indeterminate	2.2 (1.4–3.0)		4.4 (3.6–5.2)						
				(−)	2.3 (1.9–2.8)		1.7 (1.2–2.1)						
van den Bergh et al 2010 [41] N = 600–641 • Age: 50–75 years • NELSON study participants	RCT	LDCT	IES (total)	Indeterminate	4.5 (6.5)	4.9 (8.4)	8.3 (11.3)*^,c^						
				(−)	4.1 (7.4)	4.5 (7.7)	2.4 (5.5)*^d^						
			IES-intrusive	Indeterminate	2.0 (3.0)	2.0 (3.8)	3.5 (5.2)						
				(−)	1.7 (3.5)	1.8 (3.5)	0.8 (2.4)						
			IES-avoidance	Indeterminate	2.5 (4.1)	2.9 (4.9)	4.8 (6.9)*^c^						
				(−)	2.4 (3.7)	2.7 (4.7)	1.5 (3.7)						
van den Bergh et al 2008 [43] N = 288–324 • Age: 50–75 years • NELSON study participants	RCT	LDCT	IES (total)	Total (−/Incidental)	3.0^e^	1.0^e^							2.0^e^
			IES-intrusive		1.0^e^	0.0^e^							0.0^e^
			IES-avoidance		1.0^e^	0.0^e^							1.0^e^
Bunge et al 2008 [49] *N* = 40–47 (High AR) *N* = 236–247 (Low AR) • Age: 50–75 years • NELSON study participants	RCT	LDCT	IES (total)	High AR	11.5^e^						6.5^e,f,^*		
				Low AR	2.0^e^						1.0^e,^*		
			IES-intrusive	High AR	5.0^e^						3.5^e,^*		
				Low AR	1.0^e^						0.0^e,^*		
			IES-avoidance	High AR	5.0^e^						3.0^e,^*		
				Low AR	1.0^e^						0.5^e,^*		

*Indicating statistical significance, *p* < 0.05

^†^IES, IIRS: higher scores indicate more distress

^a^Levels of distress decreased upon receipt of normal scan results (*p* = 0.007), when compared to after screening

^b^Clinically relevant (MID = half an SD) and significantly higher levels of distress from baseline to 2 months in indeterminate group, compared to the (−) group

^c^Clinically relevant (MID = half an SD) and significantly higher levels of distress (MID = half an SD) from baseline to 2 months in indeterminate group, compared to 1 day after screening and before baseline

^d^Clinically relevant (MID = half an SD) and significantly lower levels of distress (MID = half an SD) from baseline to 2 months in (−) group, compared to 1 day after screening and before baseline

^e^Median values

^f^*p* < 0.01 versus low AR

*Abbreviations*: *AR* affective risk, *CA-125* ovarian tumor marker, *IES* impact of event scale, *LDCT* low-dose computed tomography, *NELSON* The Dutch-Belgian Randomized Lung Cancer Screening Trial, *RCT* randomized controlled trial, *SD* standard deviation, *TVS* transvaginal ultrasound scan

**Table 4:** Patient-Reported Outcomes Related to Worry

	Study Design	Screening	Measure^†^	Result	Screening		2 mo	3 mo	4 mo	5 mo	6 mo	7 mo–11 mo	12 mo
					Baseline	Within 1 mo							
					Mean (SD)	Mean (SD)	Mean (SD)						
Brain et al 2016 [37] N = 1579–2019 N = 73 (−) N = 41 (Incidental) N = 788 (+)/Repeat N = 48 (+)/MDT	RCT	LDCT	CWS	Total (+/−/Incidental)	8.8	8.5 (8.4–8.6)							
				(−)		8.3 (8.2–8.5)							
				Incidental		8.6 (8.0–9.2)							
				(+)/Repeat scan		9.3 (9.2–9.5)*							
				(+)/MDT referral		11.9 (11.1–12.7)*							
				Control	8.7	8.3 (8.2–8.4)							
Ong et al 2016 [50] *N* = 234–327	Prospective questionnaire	DARE	Cancer worry	Total (regardless of result)	NA	• Worried about dying soon: 4% • Thought about anal cancer a lot: 3% • Worried about developing anal cancer: 3%							
Landstra et al 2013 [51] *N* = 50–271 • HIV+ and MSM with history of (non-cancer) anal disease	Prospective longitudinal survey	Anal Swab + HRA	ASQ	(+)	≈1.3^a^	≈1.8^a^	≈1.7^a^						
				(−)	≈1.2^a^	≈1.2^a^	≈0.9^a^						
				FP	≈1.3^a^	≈1.8^a^	≈1.2^a^						
Ruberg et al 2016 [47] N = 180 (Total) N = 16 (Abnormal) • Age: 30–60 years • Women living in Netherlands	Prospective longitudinal	TVS/CA125	MWM	Total (Abnormal, Normal)	3.8 (1.7)	3.3 (1.6)	2.6 (1.5)						
				Abnormal	4.5 (1.8)	3.8 (1.7)	3.5 (1.8)						
				Normal	3.7 (1.7)	3.2 (1.6)	2.5 (1.4)*						
			CWS	Total (Abnormal, Normal)	2.1 (1.6)	1.7 (1.3)	1.3 (1.2)						
				Abnormal	2.7 (2.0)	1.9 (1.0)	2.0 (1.2)						
				Normal	2.1 (1.6)	1.7 (1.3)	1.3 (1.2)*						
Byrne et al 2008 [44] N = 341 • Age: 50–79 years	Prospective cohort	LDCT	PCQ	Suspicious	6.4 (2.3)	8.5 (2.6)*					7.4 (3.0)		7.1 (2.5)
				Indeterminate	7.2 (2.8)	7.5 (2.7)					7.1 (2.6)		7.1 (2.7)
				(−)	7.0 (2.5)	7.0 (2.4)					6.5 (2.4)		6.7 (2.3)
Tyndel 2007 [52] Recall: 112 Clear: 1174 • Age: 35–49 years • Women with a moderate or high risk of familial breast cancer	Prospective cohort	MMG	CWS	Recall	11.6 (2.9)	11.7 (2.9)					10.4 (2.7)*		
				All-clear	11.0 (2.9)	10.6 (2.6)*					10.1 (2.5)*		

*Indicating statistical significance, *p* < 0.05

^†^CWS, MWM, PCQ: higher scores indicate greater worry

^a^Estimated numbers from figure in manuscript

*Abbreviations*: *ASQ* anal screening questionnaire, *CA-125* ovarian tumor marker, *CWS* cancer worry scale, *DARE* digital anal rectal examination, *FP* false positive, *HRA* high resolution anoscopy, *LDCT* low-dose computed tomography, *MDT* multidisciplinary team, *MMG* mammogram, *MSM* men who have sex with men, *MWM* Magnitude Worry Measure, *NA* not applicable, *PCQ* Psychological Consequences Questionnaire, *RCT* randomized controlled trial, *SD* standard deviation, *TVS* transvaginal ultrasound scan

### Symptoms of anxiety

#### Measures of symptoms of anxiety

Assessment of symptoms of anxiety within lung, breast, colorectal, anal, prostate, and cervical cancer screening programs and trials was included in 17 studies [17, 34–46, 51, 53, 54] (Table 1), of which three studies [51, 53, 54] did not report specific PROM scores and were therefore not included in Table 1. In total, 9 studies [17, 34, 38, 41, 43–46, 53] used the State-Trait Anxiety Inventory (STAI), 5 studies [35–37, 39, 42] used the Hospital Anxiety and Depression Scale (HADS), and the remaining 3 studies [51, 53, 54] used the Consequences of Screening in Lung Cancer (COS-LC) questionnaire, Depression Anxiety Stress Scales (DASS), and Bowel Cancer Screening questionnaire (custom questionnaire). Of note, 8 studies [17, 34, 38, 41, 43, 45, 46, 53] used the STAI-state subscale specifically, one study [44] used both the STAI-state and STAI-trait subscales, and 5 studies [35–37, 39, 42] used the HADS-anxiety (HADS-A) subscale. Additionally, two of the lung cancer screening studies were based on the NELSON trial [41, 43] and used the STAI, and two of the colorectal cancer screening studies were based on the BCSN pilot study [35, 36] and used the HADS. For all measures administered, higher scores indicate greater anxiety.

The STAI Form Y consists of 20 items each for assessing state anxiety (i.e., current state of anxiety) and trait anxiety (i.e., trait of personality which describes tendency to present state anxiety), for a total of 40 questions [55]. Each of the state and trait anxiety subscales of the STAI is rated on a 4-point scale (not at all, somewhat, moderately so, very much so), with the range of possible scores varying from a minimum score of 20 to a maximum score of 80. STAI scores are commonly classified as “no or low anxiety” [20–34, 39, 41, 43], “moderate anxiety” [35–37, 42, 44, 46, 51], and “high anxiety” (45–80) [55]. Other studies have suggested a cutoff score of 39–40 to detect clinically significant symptoms for the state-anxiety scale, as well as a higher score of 54–55 for older adults [56].

The DASS consists of three self-report scales designed to measure the negative emotional states of depression, anxiety, and stress. Consisting of 42 items, these three scales are each comprised of 14 items, with the anxiety scale assessing autonomic arousal, skeletal muscle effects, situational anxiety, and subjective experience of anxious affect [57]. Subjects are asked to use 4-point severity/frequency scales to rate the extent to which they have experienced each state *over the past week*. Scores for Depression, Anxiety, and Stress are calculated by summing the scores for the relevant items.

The HADS consists of 14 items, or two 7-item subscales, one of which is anxiety (HADS-A) and other, depression (HADS-D). For the HADS-A, each item is scored from 0 to 3, and the maximum score is 21, with higher scores indicating higher levels of anxiety. A score of 0–7 for either subscale could be regarded as normal, 8–10 as suggestive of the presence of an anxious state, and a score of ≥11 indicating probable presence of anxiety [58, 59].

The COS-LC measures psychosocial consequences in lung cancer screening, and was developed based on the Consequences of Screening in Breast Cancer (COS-BC) questionnaire [40]. The Consequences of Screening (COS) questionnaire is a common core questionnaire of the COS-LC and COS-BC, and encompasses four scales, one of which is anxiety, with four response categories and scores: not at all (0), a bit (1), quite a bit (2), and a lot (3) [40]. The higher the score, the more negative psychosocial consequences the person has experienced.

Finally, the Bowel Cancer Screening questionnaire refers to a simple custom questionnaire containing one question about anxiety before and after the test, with possible response options being not anxious, moderate anxiety, or severe anxiety [54].

#### Results for symptoms of anxiety

Symptoms of anxiety varied considerably throughout the screening process, depending on the test result and timing with respect to the result. Across multiple studies [34, 36–38], symptoms of anxiety increased following a positive test result (approximately 1–2 weeks to 1 month after screening). Symptoms of anxiety then decreased 3–6 months after screening and persisted at 1 year after screening. A similar trend was observed following a suspicious, indeterminate, or false-positive result, with an increase in symptoms of anxiety within the first 2 months of screening [39, 41, 44]. Following a negative result, however, these symptoms tended to decrease temporarily or remain unchanged (Table 1).

##### Impact of positive screening results on symptoms of anxiety

Among participants in the United Kingdom Lung Cancer Screening trial (UKLS) who received a positive LDCT scan result (Brain et al. 2016) [37], there was a statistically significant increase in symptoms of anxiety at 2 weeks from baseline, as measured by the HADS-A. This increase was not reported as clinically significant. Similarly, those receiving a positive fecal occult blood test (FOBT) result also experienced a statistically significant increase in symptoms of anxiety at 2 weeks from baseline, followed by a decrease at 4 months (Laing et al. 2014) [38]. While these individuals with a positive FOBT reported higher levels of situational anxiety, however, the mean STAI-state score was not clinically meaningful (i.e., defined in the study as STAI-state score ≥ 54) at any time point [38]. Within the Pan-Canadian Early Detection of Lung Cancer Study, Taghizadeh et al. 2019 [34] reported a statistically significant increase in symptoms of anxiety following a positive LDCT scan at 1 month from baseline. More participants in this study had a clinically significant increase (i.e., minimal clinically important difference (MCID) > 10) in symptoms of anxiety at 1 month following receipt of positive results, and to a much lesser degree at 12 months. Conversely, in the BCSN pilot trial, Kirkoen et al. 2016 [36] reported an increase in symptoms of anxiety immediately following a positive flexible sigmoidoscopy or fecal immunochemical test (FIT) from baseline, followed by a decrease at 6 and 12 months. However, these findings were not statistically or clinically significant.

##### Impact of abnormal or false-positive screening results on symptoms of anxiety

In the Pittsburgh Lung Screening Study, Byrne et al. 2008 [44] found a statistically significant increase in state anxiety 1–2 weeks after receipt of indeterminate LDCT scan results, despite returning to baseline at 12 months. Conversely, the sample size for those receiving suspicious results was small and these individuals’ state anxiety did not significantly change over time while their trait anxiety increased only slightly at 1–2 weeks [44]. In the NELSON trial by van den Bergh et al. 2010 [41], individuals with indeterminate LDCT scan results reported a statistically significant increase in symptoms of anxiety at 2 months from baseline using the STAI-state, compared to those receiving negative results. These differences, however, were not clinically meaningful (i.e., they did not exceed the MCID, or half of a standard deviation of the mean). Within the same trial, van den Bergh et al. 2008 [43] found a temporary decrease in symptoms of anxiety from baseline to 1 day post-LDCT scan and prior to receiving results, followed by a return to baseline at 6 months in individuals with indeterminate or negative results, though changes were minimal and smaller than the MCID.

In a separate study by Hafslund et al. 2012 [39], individuals with a false-positive result from a mammogram had a temporary but statistically significant increase in symptoms of anxiety at 2 weeks compared to baseline, followed by a decrease at 6 and 12 months. Of those individuals, 23 women (18%) reported a clinically significant level of anxiety (i.e., HADS-A score ≥ 8) at 2 weeks [39]. Conversely, in women from hereditary nonpolyposis colorectal cancer families undergoing screening, Wood et al. 2008 [42] did not observe any changes in their symptoms of anxiety at 3 and 6 months from baseline, even following false-positive results.

##### Impact of normal screening results on symptoms of anxiety

In contrast, in the BCSN pilot study, Kirkoen et al. 2016 [35] reported a statistically significant decrease in symptoms of anxiety following receipt of negative flexible sigmoidoscopy or FIT results in participants. In addition, Korfage et al. 2012 [17] reported decreased general anxiety based on the STAI-state in individuals with normal Pap smear results while Cormier et al. 2002 [46] reported a moderate increase in symptoms of anxiety shortly after screening in approximately 20% of individuals with normal PSA test results. Of note, in Cormier et al. 2002 [46], while there was a statistically significant increase in symptoms of anxiety in individuals with normal results, there were fewer individuals with a clinically significant increase in symptoms of anxiety (i.e., MCID = 1 standard error of measurement) after receiving normal results compared to the number of individuals with an increase in symptoms of anxiety prior to receipt of results. As shown in Table 1, the STAI-state scores have been standardized to a scale of 0 (poorest status) to 100 (best status) [46]. In addition, in a study by Williams et al. 2006 [54], approximately 84% of asymptomatic relatives of family members with colorectal cancer reported having moderate or severe anxiety before their colonoscopy on the Bowel Cancer Screening Questionnaire. Nearly 42% of individuals still reported having anxiety after undergoing their colonoscopy and receiving normal results (results not reported in Table 1). Finally, minimal differences in symptoms of anxiety were reported in Aggestrup et al. 2012 [40], using the COS-LC, at 1 year from baseline in individuals with normal LDCT results in the Danish Lung Cancer Screening Trial.

##### Impact of nonspecific screening results on symptoms of anxiety, overall and within subpopulations

Regardless of screening test results, a study by Robb et al. 2012 [53] demonstrated no changes in symptoms of anxiety, as measured by the STAI-state, 3 months after receiving flexible sigmoidoscopy compared to baseline, though over 25% reported clinically significant symptoms of anxiety (i.e., STAI-state score > 44) at 3 months (results not reported in Table 1). Similarly, in Landstra et al. 2013 [51], there were no differences in symptoms of anxiety using the DASS in individuals receiving anal swab and high-resolution anoscopy, regardless of test result (results not reported in Table 1).

In the lung cancer screening study by Byrne et al. 2008 [44], state anxiety reportedly increased in current smokers and decreased in those with higher levels of education, regardless of test result. These changes in symptoms of anxiety were statistically significant, but not indicated as clinically significant. There was also a statistically significant increase in symptoms of anxiety in females undergoing LDCT, those concerned about getting lung cancer at baseline [34], and in females receiving positive flexible sigmoidoscopy or FIT results [36]. Finally, in the study by Absetz et al. 2003 [45], compared to those with low perceived susceptibility (i.e., low perceived risk of cancer), individuals with high perceived susceptibility had elevated STAI-state scores after a mammogram (two months from baseline) before dropping slightly at 12 months. Changes were not statistically or clinically significant.

### Symptoms of depression

#### Measures of symptoms of depression

Seven studies [35–37, 39, 42, 45, 51] included an assessment of symptoms of depression in screening programs for lung, breast, colorectal, and anal cancer (Table 2). One study [51] did not report any PROM scores and was not included in Table 2.

These studies implemented 3 measures of depression, including the HADS, Beck’s Depression Inventory (BDI), and DASS. Of the 7 studies, 5 studies [35–37, 39, 42] used the HADS-depression subscale (HADS-D), a 7-item subscale that measures depressive mood and symptoms with a 1 week recall period [59], and a cutoff score of 8 indicating possible presence of depression [35, 36]. One study [45] used the BDI-I, which is a 21-item measure that assesses the characteristic symptoms of depression [60], with cutoff scores of 0–9 indicating no or minimal depression, and 10–18 indicating mild-to-moderate depression. The final study [51] used the depression subscale of the DASS, which consists of 14-items that assess dysphoria, hopelessness, devaluation of life, self-deprecation, lack of interest/involvement, anhedonia, and inertia [57]. On all measures, higher scores indicate more symptoms of depression.

#### Results for symptoms of depression

There were minimal-to-moderate changes in symptoms of depression or mood across all studies, as shown in Table 2. Symptoms of depression were greater following positive and false-positive test results 2 weeks post screening [37, 39]. They were also more pronounced and less transient in women [36, 45].

##### Impact of positive screening results on symptoms of depression

In the UKLS study, Brain et al. 2016 [37] reported an increase in HADS-D score in individuals with a positive LDCT scan requiring referral to the multidisciplinary team 2 weeks post baseline, compared to those receiving negative results, incidental findings, or positive results requiring a repeat scan, though this difference in scores was neither statistically nor clinically significant. Alternatively, in the BCSN pilot study by Kirkoen et al. 2016 [35, 36], no clinically relevant changes in symptoms of depression (i.e., half of a standard deviation of the mean) were documented in individuals receiving a positive flexible sigmoidoscopy or FIT result 12 months post baseline, based on the HADS-D. However, symptoms of depression were observed in a subgroup of women immediately after receiving a positive flexible sigmoidoscopy or FIT result (*p* < 0.01), but to a much lesser extent in men immediately after a negative flexible sigmoidoscopy or FIT result (*p* < 0.01). None of these changes fulfilled the criteria of clinically relevant change (i.e., half of the standard deviation or Cohen’s d above 0.5).

##### Impact of false-positive screening results on symptoms of depression

Following receipt of false-positive test results, differences in levels of depressed mood were observed. In one study by Hafslund et al. 2012 [39], participants in a breast cancer screening trial reported higher levels of depressed mood on the HADS-D at 2 weeks after receiving false-positive results from a mammogram compared to baseline, and scores remained elevated 3 and 6 months after screening. Scores were significantly higher in these women receiving false-positive results at 6 months (*p* = 0.045), compared to those with negative results. In contrast, Wood et al. 2008 [42] observed no clinically relevant mean changes in depressed symptoms on the HADS-D at 3 or 6 months after false-positive gynecological screening in women from hereditary nonpolyposis colorectal cancer families.

##### Impact of nonspecific screening results on symptoms of depression, overall and within subpopulations

Regardless of screening test results, differences in levels of depressed mood were observed in one breast cancer screening study by Absetz et al. 2003 [53], which included women with varying levels of breast cancer risk and a baseline perceived susceptibility to breast cancer. Women with a high perceived susceptibility reported a temporary decrease in levels of depressed mood on the BDI-I 2 months after screening before reverting back to baseline levels at 12 months. The level of depressed mood among women with high perceived susceptibility was not clinically significant. In contrast, no significant changes in symptoms of depression were detected from baseline (i.e., pre-screening) to post-screening assessment timepoints among the remaining three lung, colorectal, and anal cancer screening studies. Similarly, regardless of test results for flexible sigmoidoscopy or FIT in the BCSN pilot study by Kirkoen et al. 2016 [35, 36], or for LDCT in the UKLS by Brain et al. 2016 [37], there were no clinically relevant changes in symptoms of depression from baseline. Also, there were no statistically or clinically significant changes observed on the DASS from baseline to receiving anal cancer screening results according to Landstra et al. 2013 [51] (results not reported in Table 2).

### Distress

#### Measures of distress

Seven studies [41, 43, 47–49, 61, 62] included an assessment of distress, including intrusive thoughts, in screening programs and trials for lung, anal, prostate, cervical, and ovarian cancer (Table 3). Two studies [61, 62] did not report specific PROM scores and were therefore not included in Table 3. Also, three of the lung cancer screening studies were based on the NELSON trial and used the Impact of Events Scale (IES).

The most commonly used measure of subjective distress was the IES (*n* = 6), which consists of 15 questions with scoring of 0–75 (> 26 indicating moderate-to-severe impact) that measure the amount of distress associated with a specific event or life stressor, and consists of two subscales that measure intrusion (scored 0–35) and avoidance (scored 0–40) [63]. Levels of distress may also be classified as low (< 8.5 on either the avoidance or intrusion subscales of the IES), medium [9–19], or high (> 19) [62]. The higher the score, the greater the distress.

Finally, the Illness Intrusiveness Ratings Scale (IIRS) is comprised of 13 items, with each grading the extent to which illness interferes with that domain by use of a 7-point (ranging from 1, not very much, to 7, very much) scale [62]. Scores range from 7 to 51, but the exact clinical interpretation is unknown. As such, elevated levels of negative impact were defined a priori, as either 1) a score of 7 (highest level of intrusion) on ≥1 of the 13 items in the scale or 2) a score ≥ 3 on ≥3 of the 13 items [62].

#### Results for distress

Across most of the studies, levels of distress increased in individuals shortly after receiving indeterminate or abnormal results [41, 47, 61], and in those with a family history of cancer and/or a higher affective risk perception [49, 61], both of which may collectively prolong distress for up to 6 and 12 months, as shown in Table 3.

##### Impact of indeterminate or abnormal screening results on distress

Among those receiving indeterminate LDCT scan results in the NELSON trial by van den Bergh et al. 2010 [41], lung cancer-specific distress increased in a statistically significant manner at 2 months, compared to before the baseline scan and 1 day after screening. Only the changes in IES scores between baseline and 2 months were clinically significant (i.e., minimal important difference (MID) = half of a standard deviation). Similarly, van den Bergh et al. 2011 [48] observed an increase in total IES scores (worsening distress) at 2 months in individuals with indeterminate results from baseline. However, only the differences between those with indeterminate results and those with negative results exceeded the MID at 2 months and were clinically relevant.

Several studies reported levels of distress among individuals with abnormal results. Taylor et al. 2004 [61] found that participants in the Prostate, Lung, Colorectal, and Ovarian (PLCO) cancer screening arm with abnormal screening results had a statistically significantly higher level of intrusive thoughts about cancer than those with normal screening results at 4–8 weeks (66% vs 44% of patients, *p* = 0.03) and 12 months after screening (66% vs 53% of patients, *p* = 0.03), though this was not indicated as clinically significant. Of note, 45% of individuals reported levels of distress at baseline (results not reported in Table 3). In Ruberg et al. 2016 [47], participants in the University of Louisville Ovarian Cancer Screening Study reported an increase in intrusive thoughts from baseline to up to 2 months after screening for individuals receiving abnormal results. Levels of distress were not indicative of clinically significant distress, however.

##### Impact of normal screening results on distress

Among those with negative screening test results, distress levels decreased statistically significantly at 2 months following an LDCT scan, compared to before baseline scan and 1 day after screening in two previous studies by van den Bergh et al. 2008 and 2010 [41, 43]. In both studies, the reported changes in IES scores were smaller than the MID (i.e., half of a standard deviation) and not clinically significant. A decrease in distress levels was also seen in van den Bergh et al. 2011 [48], though not significant. Ruberg et a 2016 [47] also observed a statistically significant decrease in levels of distress upon receipt of normal scan results at 14–30 days after screening, compared to before the baseline scan.

##### Impact of nonspecific screening results on distress, overall and within subpopulations

Regardless of results, median IES scores were statistically significantly lower at 6 months after an LDCT scan from baseline within both high and low affective risk perception groups (all *p* < 0.05) in Bunge et al. 2008 [49]. However, participants with a high affective risk perception still reported significantly higher IES scores at 6 months, than those with a low affective risk perception, though the levels of distress were not severe or clinically significant (e.g., MID = half of a standard deviation). Within the low affective risk group were those that felt their risk was very low or low, who did not show a lower median total IES score at 6 months after screening. Conversely, those who felt their risk was not low/not high, showed a statistically significantly lower median total IES score 6 months after screening compared to 1 day before screening (3.0 vs 2.0, *p* < 0.01) [49]. Additionally, regardless of result type, Tinmouth et al. 2011 [62] observed that human immunodeficiency virus (HIV)-infected men who have sex with men in the Toronto Research for Anal Cancer Evaluation (TRACE) study tended to have elevated levels of distress (IES score ≥ 9 on either the intrusiveness or avoidance subscales) within 1 week of screening (29% vs 22%), but less so after receiving results (24%) and at 6 months (25%), based on the IES. Similar trends were seen using the IIRS (elevated IIRS score = 9 on ≥1 items in the scale, or score ≥ 3 on ≥3 of the items) within 1 week of screening (32% vs 25%) and at 6 months (15%) (results not reported in Table 3).

Finally, levels of distress were significantly higher in current smokers [41] (*p* < 0.01) and in those who found waiting for the CT scan result to be discomforting (*p* < 0.01) in Bunge et al. 2008 [49]. Also, there was a temporary increase in levels of distress 1 week after anal cancer screening in younger individuals (*p* = 0.02), those with more HIV-related symptoms (*p* = 0.008), and those with a greater baseline psychological distress (*p* < 0.0001) [62]. In the PLCO cancer screening study, Taylor et al. 2004 [61] reported levels of distress were significantly higher at 4–8 weeks from baseline in females (*p* = 0.04) and in individuals with a first-degree relative with cancer (*p* = 0.01).

### Worry

#### Measures of worry

Eight studies [37, 38, 44, 47, 50–52, 64] included an assessment of cancer worry, including fear of cancer, in screening trials for lung, breast, colorectal, anal, and ovarian cancer (Table 4). Three studies [38, 50, 64] did not report specific PROM scores, and were therefore not included in Table 4.

The most common measure was the Cancer Worry Scale (CWS; *n* = 3), including the revised versions of the CWS. The CWS is anchored to thoughts and feelings about cancer during past week (total score ranges from 6 to 24 with scores from 1 (not at all or rarely) to 4 (almost all of the time) for the 6-item measure) and assesses the impact of these concerns on mood and daily functioning [37, 52]. In the UKLS [37], scores > 12.5 on the CWS corresponded to a clinically significant threshold score on the GHQ-28. In the University of Louisville Ovarian Cancer Screening Study [47], the 3-item CWS was modified and used to assess ovarian cancer worry, frequency of worrisome thoughts, and impact of worry on mood and daily functioning, with scores ranging from 0 to 13.

The Psychological Consequences Questionnaire (PCQ) assesses the emotional, social, and physical consequences of breast cancer screening, with higher total scores indicating greater fear of cancer [65]. One study measured worry using three questions adapted for the PCQ to assess the effects of screening on fears of lung cancer [44]. The Magnitude of Worry Measure (MWM), consists of a single-item measure assessing magnitude of worry on a seven-point Likert-type scale with the following anchors: 1 = not at all worried and 7 = extremely worried [47].

Additionally, colorectal cancer-specific worry was measured by two single-item questionnaires measuring worry frequency and recent colorectal cancer-specific worry on mood, both rated on a 5-point Likert scale ranging from 1 = never to 5 = all the time. Respondents were designated as having “no worry” or “no impact on mood” by combining the following results (1 = never and 2 = rarely), and “yes, at least some worry/impact on mood” by combining the following results (3 = sometimes, 4 = most of the time, and 5 = all of the time) [38]. In a separate study, a different worry questionnaire was developed and administered at a single time point to assess how worried participants felt at various stages of the screening mammogram process, using a 4-point Likert scale ranging from “not at all,” “a little bit,” “quite,” and “very.” [52] Participants undergoing digital anal rectal examination were also given a survey, answering questions regarding cancer worry with such responses as “thought about anal cancer a lot” and “worry about developing anal cancer.” [50]

The Anal Screening Questionnaire (ASQ) was modified from the Cervical Screening Questionnaire and used in one study [51], where the worry questions from the Cervical Screening Questionnaire were omitted, and the adapted prostate cancer worry items from McNaughton Colins et al. [66] were used, to measure worry about cancer, dying soon, and reassurance from testing.

#### Results for worry

There was a temporary increase in fear of cancer or cancer worry shortly after screening or after receiving indeterminate, abnormal, or suspicious results [37, 38, 44, 52, 64], though these effects dissipated after 3 months (Table 4). Significant increases in worry were also seen among females, current smokers, younger individuals, and in one New Zealand study of Maori and Pacific Island women.

##### Impact of positive screening results on worry

Among those who received a positive FOBT result, more individuals reported experiencing an increase in worry frequency (35% vs 18%) and mood disturbances (21% vs 4%) at 1–2 weeks post screening (within 2 days of the result), compared to baseline in Laing et al. 2015 [38]. Worry frequency persisted at 4 months post results in 29% of individuals, whereas mood disturbances were present in only 5% of individuals (results not reported in Table 4). In Brain et al. 2016 [37], participants in the UKLS who received positive LDCT results reported statistically significantly higher levels of worry at 2 weeks with the CWS, compared to baseline. This increase in worry did not reach a clinically significant threshold score (e.g., CWS score > 12.5). In a separate study by Landstra et al. 2013 [51], HIV-positive individuals and men who have sex with men with positive high-resolution anoscopy results also had a statistically significant increase in worry as reported by the ASQ at 2 weeks and 8–10 weeks, compared to those with normal results, though not clinically significant. Levels of worry were also higher at both time points, compared to baseline, though also not statistically or clinically significant.

##### Impact of suspicious screening results on worry

One study reported levels of worry among those receiving suspicious results. Byrne et a 2008 [44] observed a statistically significant increase in fear of cancer at 1–2 weeks after receiving suspicious results from an LDCT scan from baseline. This increase in fear persisted at 6 and 12 months after screening, using the PCQ.

##### Impact of false-positive screening results on worry

Among individuals receiving false-positive results, HIV-positive individuals and men who have sex with men reported a statistically significant increase in worry at 2 and 8–10 weeks, compared to those with normal results in Landstra et al. 2013 [51]. Also, compared to baseline, worry levels increased temporarily at 2 weeks before returning to baseline at 8–10 weeks. These results were not indicated as clinically significant, however. Conversely, in Tyndel et al. 2007 [52], levels of worry decreased at 6 months in false-positive/recall group from baseline, and women receiving a false-positive result did not show a statistically significant increase in cancer worry after receiving their results.

##### Impact of normal screening results on worry

Among those receiving negative screening results, Laing et al. 2015 [38] observed few individuals receiving negative FOBT results report worry or mood disturbances, and this did not change over time (results not reported in Table 4). Those with negative LDCT results in Brain et al. 2016 [37] reported a slight decrease within 2 weeks post-exam, but this was not statistically or clinically significant.

##### Impact of nonspecific screening results on worry, overall and within subpopulations

Regardless of screening test results, HIV-positive men who have sex with men, and who are undergoing digital anal rectal examination, reported the overall experience to be positive and acceptable. Approximately 3% of men reported thinking more about anal cancer and were worried about developing anal cancer and about dying soon in Ong et al. 2016 [50] (results not reported in Table 4). Similarly, Tyndel et al. 2007 [52] observed a statistically significant decrease in worry levels at 1 and 6 months using the CWS in those with normal results, compared to baseline. Clinical significance was not reported. Furthermore, in Brunton et al. 2005 [64], a notable increase in individuals reporting worry while awaiting their mammography appointment and results from baseline (18% vs 11%), but approximately 67% reported experiencing reassurance some months following receipt of clear results (results not reported in Table 4). However, levels of worry on the MWM and CWS were highest prior to screening in Ruberg et al. 2016 [47], before decreasing significantly at 1 month after screening and receiving a normal result Byrne et al. 2008 [44], the average fear of cancer scores for those with negative screens stayed fairly level over time.

Worry and fear of lung cancer increased significantly in females [37, 44] (*p* < 0.03), current smokers [37, 44] (*p* < 0.001), those with lower levels of education [44] (*p* < 0.03), and those aged ≤65 years [37] (*p* ≤ 0.001). Similarly, a statistically significant increase in worry about breast cancer was seen in those with a lower education (*p* = 0.018), family history of breast cancer (*p* = 0.002), stress levels during screening mammography (*p* < 0.001), and experience of pain during the procedure (*p* < 0.001) [64], as well as Maori and Pacific Island women (*p* < 0.001), though it is unclear whether these changes are clinically significant.

### Other functional status and well-being, preference-weighted health status, and other psychosocial

#### Measures of, and results for, other functional status and well-being, preference-weighted health status, and other psychosocial

The remaining three constructs included functional status and well-being, preference-weighted health status, and other psychosocial.

The majority of studies [17, 34–36, 39, 41–43, 46, 48–51, 61, 67–71] (*n* = 19) included an assessment of functional status and well-being, with the most common measure being the 12-Item Short Form Survey (SF-12), which is one of the most widely used generic health status instruments for assessing self-reported health-related quality of life (HRQOL) with standardized scores (i.e., mean, 50; standard deviation, 10) [36]. Overall, minimal changes in functional status and well-being were observed in either the short- or intermediate-term post-screening period. Significant decreases in functional status and well-being were seen in a subset of individuals receiving abnormal or positive test results as well as in women, those aged < 64 years, and those with a high affective risk perception at baseline. A summary of the results can be found in Additional file 7: Table S7.

Similarly, 7 studies [17, 34, 41, 43, 48–50] assessed preference-weighted health status. The most commonly used measure was the EQ-5D, which is generally calibrated with preferences from the whole population in one country to classify general HRQOL (mobility, self-care, usual activities, pain/discomfort, anxiety/depression) and quantify patients’ self-rated health [72]. In some studies, respondents were also asked to rate their own health on the EQ-5D visual analog scale, ranging from 0 (worst imaginable health status) to 100 ﻿(best imaginable health status) [72–74] as well. Additionally, two studies reported Short-Form Six-Dimension (SF-6D) utility scores, which were either derived from the SF-36 using the UK scoring algorithm [67], or the SF-12 using the University of Sheffield’s SF-6D classification for describing health [50]. The SF-6D estimates a preference-based single index measure for health using general population values, and allows utility scores to be obtained with scores covering a range of 0 (worst health state) to 1 (best health state) [40, 53]. Across all cancer screening studies, changes in preference-weighted health status were minimal over time, though a temporary decrease in health status was reported shortly after receiving abnormal or indeterminate results. However, scores returned to baseline shortly thereafter. A summary of the results can be found in Additional file 8: Table S8.

Finally, 13 studies [17, 37, 40, 43–45, 49, 50, 52, 53, 61, 67, 75] included assessment of other psychosocial measures, including satisfaction with the screening exam or decision to participate in the trial, discomfort, perceived risk of developing cancer, and general psychosocial consequences, in screening studies for lung, breast, colorectal, anal, prostate, ovarian, and cervical cancer (Additional file 9: Table S9). Overall, individuals’ satisfaction with the exam and decision to participate in the screening trial was high and the experience with the screening process was positive, with minimal levels of discomfort experienced while waiting for the test results. Individuals varied widely with respect to their perceived risk of developing cancer, but those with a higher perceived susceptibility experienced more negative psychosocial consequences.

## Discussion

Our review found there was a temporary increase in symptoms of anxiety, distress, and worry around 2 weeks to 1 month after the cancer screening test, compared to before the test (i.e., baseline) [34, 37, 38, 43, 44]. This finding was more prominent in individuals with an indeterminate or false-positive screening result. However, no long-term psychosocial consequences were detected in individuals with indeterminate or false-positive results. Also, as expected, individuals with negative results generally reported fewer symptoms of anxiety and better functional status and well-being. More symptoms of anxiety were reported immediately after screening [62], while waiting for the screening results [68], and with more invasive screening tests (e.g., flexible sigmoidoscopy compared to the FIT) [35, 36].

Our review also had some unexpected findings. In women younger than 60 years with a family history of breast cancer who were recalled for additional tests as a result of a positive or abnormal mammogram, levels of cancer-specific distress did not change. Instead, these individuals reported more positive psychological consequences at 1 month, compared to those who received negative cancer screening test results. Although this effect was not seen at 6 months, the additional follow-up may temporarily decrease levels of distress due to the reassurance and beliefs in the benefits of screening [52]. The positive perceptions of screening suggest that the women had different expectations of screening and viewed distress caused by additional testing as an acceptable part of screening. In a separate study, while anxiety levels dissipated in women with false-positive results from a mammogram following diagnostic resolution, more symptoms of depression were reported at 6 months [39]. This may have resulted from an overall increase in distress and intrusive thoughts among women with false-positive results [39], which may have interrupted their normal daily activities and made them feel they are less healthy than others. Finally, in a cohort of individuals with negative LDCT scan results, sustained reduction in fear of cancer after receipt of results was not reported, suggesting negative screening results may not result in persistent reduction with regard to fear of cancer. In fact, these individuals may have realized that screening results are subject to change and regular testing is still needed.

Specific subpopulations (e.g., those with a family history of cancer or aged ≥50 years, females, current smokers) with an elevated risk of cancer may have different expectations of screening which may alter the impact of screening in these individuals. For instance, those with a strong family history of cancer (e.g., a first-degree relative with cancer) may have different levels of anxiety, by nature of a higher perception of their own risk of developing the disease, compared to those with no family history of cancer. These individuals reported higher levels of worry about breast [64] or lung cancer [44], higher levels of distress and intrusive thoughts about cancer [61], appreciable and persistent levels of anxiety after a mammography [45] or even a normal colonoscopy [54], and decreased functional status and well-being outcomes [46, 61]. Furthermore, regardless of screening test result, women reported significantly higher levels of anxiety [34, 36, 41, 44], worry, and distress [37, 44, 61, 64], and poorer functional status and well-being [34] compared to men, and older individuals, particularly those aged ≥50 years had worse HRQOL [46], compared to those aged < 50 years. Similarly, current smokers reported more lung cancer-specific distress and significantly more worry, compared to nonsmokers [44] or former smokers [37, 41], regardless of test result. HIV-infected individuals reported higher cancer-specific worry in general from screening and adverse effects on screening-specific psychosocial measures among men with abnormal results. Finally, as recent research indicates, race and ethnicity may contribute to the development and survival rates for some cancers; in one New Zealand study, Maori and Pacific Island women reported higher levels of worry about developing breast cancer than New Zealand European and Asian women [64], suggesting there are certain ethnic subgroups that may have significantly higher levels of worry about both breast cancer and some aspects of screening mammography.

Across all studies, the most commonly assessed constructs were symptoms of anxiety and functional status and well-being, using the STAI and SF-12, respectively, followed by distress, using the IES. Overall, the majority of studies did not report a statistically significant change in these outcomes, and if the study did, the changes were small and of questionable clinical significance. This is an important finding, as this may in part be due to the sensitivity of PROMs, as measures such as the SF-12, EQ-5D, and HADS have not been primarily developed for measuring changes related to cancer screening.

The findings of this review were consistent with other review findings. Of note, very few studies report PROs for multiple cancer screening approaches, and most studies are cross-sectional and report outcomes at a single time point only. One review [76] assessed short-term (2 weeks before to 1 month after screening) screening-associated psychosocial distress, which encompassed anxiety, worry, subjective stress, and fear of cancer screening and diagnosis. The reviewers found, on average, consistently low levels of distress throughout the entire screening process [76].

To our knowledge, our review reflects a comprehensive assessment of PROMs in the context of cancer screenings published to date, across cancer types and at different time points within the cancer screening process. By identifying the key concepts and outcomes that are measured in different screening programs and trials and how they are impacted, we have laid the groundwork for constructing a framework for a conceptual model, which would inform a more standardized approach to measuring the psychosocial impact of different cancer screening types.

Limitations of this review include limited sample size in several studies in addition to the reporting of selected subgroups of individuals with differing access to health care and financial implications for a cancer diagnosis, all of which may limit generalizability. In addition, there may be inherent selection bias with regard to individuals participating in screening studies, as these individuals may be more motivated to receive screening (i.e., worried well) and may have better mental health. Furthermore, the lack of racial and ethnic diversity in large screening trials may also limit the generalizability of the results. Finally, this review did not systematically assess the measurement properties within the studies. Our focus was on the measures implemented and impact of cancer screening on these measures. Some of the results, however, could be affected by the measurement properties of the instruments used.

Based on the findings of this review, there are several implications to consider when assessing the psychosocial impact of cancer screening. The most relevant concepts to measure include symptoms of anxiety, distress, and worry in the short-term period (i.e., before screening and within 1 month following the screening test), and general psychosocial effects or consequences in the intermediate-term period (i.e., before screening, 6 and 12 months after screening). These domain-specific measures of psychosocial concepts which are more proximal to the actual psychological phenomena had a greater impact from screening, compared to the more distal concepts of functioning and overall well-being. These psychosocial constructs might well be tested with more specific attribution to cancer screening, though the results would be more difficult to interpret across studies. The level of specificity in assessment is unknown and is an area for future study. Future work with the Cochrane PRO Methods Group [77] can build toward a standardized approach to establish the necessary elements in future related clinical trials to permit aggregation of results. Additionally, the CONSORT-PRO guidelines [78] should be followed in future studies when reporting PROs in clinical trials.

The timing of PRO assessments is also crucial. Measures of anxiety and distress, for instance, need to be assessed in a timely manner (i.e., within 1 month after screening or receiving results) to ensure the relevant concepts and any beneficial effects or harms are in fact due to the screening exam or results. This will also help identify relevant differences between different results (e.g., negative, indeterminate). Assessing these outcomes at different timepoints for up to 1 year can also help ascertain the durability of any effect from screening.

The generalizability of results within screening trials can be enhanced in a few ways: having a large enough sample size at baseline to ensure representation of participants with positive results to enable investigation of changes within this group and having a more racially and ethnically diverse group of participants.

Finally, based on the relevant concepts and outcomes identified within this review, a future study to systematically assess the measurement properties including content validity of the identified PROMs should be conducted.

Findings from this study provide implications for good clinical practice, particularly in the primary care setting, as it is crucial to develop and implement adequate communication and education strategies to inform individuals on what to expect during and immediately after screening, as seen in the setting of genetic counseling. Provider-patient communication regarding screening tests is vital, and providers can help alleviate patients’ stress and even improve overall screening uptake by serving as a key information source [79], and coordinating screening tests and follow-up care with a clear course of action (i.e., timing of results, downstream probabilities and treatment options) based on different screening results. By keeping patients well-informed of the entire screening process, including before, during, and after undergoing screening and receiving their results, they may be more inclined to undergo screening regularly, thereby optimizing their likelihood of treatment and survival with earlier cancer detection.

## Conclusions

The psychosocial impact of cancer screening is low throughout the entire screening process, regardless of the specific cancer screening test and test result. No long-term negative psychosocial effects were observed, even in individuals with indeterminate or false-positive results. However, some higher-risk groups, such as current smokers and those with a family history of cancer, reported higher levels of distress and more symptoms of anxiety during the screening process.

Further research using measures that assess constructs including symptoms of anxiety, symptoms of depression, and worry in the context of cancer screening is needed in order to understand the relative impact of cancer screening. More attention should also be directed toward higher-risk individuals undergoing screening.

## Supplementary Information

**Additional file 1: Table S1.** Preferred Reporting Items for Systematic Reviews and Meta-Analyses (PRISMA) Checklist.

**Additional file 2: Table S2.** MEDLINE Search Strategy.

**Additional file 3: Table S3.** EMBASE Search Strategy.

**Additional file 4: Table S4.** Full Data Extraction.

**Additional File 5: Table S5.** USPSTF Quality Rating Table for Randomized Controlled Trials.

**Additional File 6: Table S6.** NIH Quality Assessment Tool for Observational Cohort Studies.

**Additional file 7: Table S7.** Patient-Reported Outcomes Related to Functional Status and Well-Being.

**Additional file 8: Table S8.** Patient-Reported Outcomes Related to Preference-Weighted Health Status.

**Additional file 9: Table S9.** Patient-Reported Outcome Related to Other Psychosocial.

## Data Availability

The datasets supporting the conclusions of the current study are included within the article and its additional files. This review has not been registered and a review protocol has not been prepared.

## Abbreviations

ASQ: Anal Screening Questionnaire
BCSN: Bowel Cancer Screening in Norway
BDI: Beck’s Depression Inventory
CONSORT-PRO: Consolidated Standards of Reporting Trials – Patient-Reported Outcomes
COS-BC: Consequences of Screening in Breast Cancer questionnaire
COS-LC: Consequences of Screening in Lung Cancer questionnaire
CWS: Cancer Worry Scale
DASS: Depression Anxiety Stress Scales
GHQ: general health questionnaire
HADS: Hospital Anxiety and Depression Scale
HADS-A: HADS Anxiety Subscale
HADS-D: HADS Depression Subscale
HIV: Human immunodeficiency virus
HRQOL: Health-related quality of life
IES: Impact of Events Scale
IIRS: Illness Intrusiveness Ratings Scale
LDCT: Low-dose computed tomography
MCID: Minimal clinically important difference
MID: Minimal important difference
MWM: Magnitude of Worry Measure
NELSON: Dutch-Belgian Lung Cancer Screening trial
NIH: National Institutes of Health
PCQ: Psychological Consequences Questionnaire
PLCO: Prostate, Lung, Colorectal, and Ovarian
PRISMA: Preferred Reporting Items for Systematic Reviews and Meta-Analyses
PRO: Patient-reported outcome
PROM: Patient-reported outcome measure
PSA: Prostate-specific antigen
RCT: Randomized controlled trial
SF-12: 12-item short-form survey
SF-36: 36-item short-form survey
SF-6D: Short-Form 6-dimension
STAI: State-Trait Anxiety Inventory
TRACE: Toronto Research for Anal Cancer Evaluation
UKLS: United Kingdom Lung Cancer Screening
US: United States
USPSTF: United States Preventive Services Task Force
