# Supplementary material for: Patient-reported outcomes associated with cancer screening: a systematic review
Source: BMC Cancer. 2022 Mar 1;22:223. doi: 10.1186/s12885-022-09261-5 (PMC8886782; doi:10.1186/s12885-022-09261-5)
Supplement: Supplementary file 2 — Additional file 2: Table S2. MEDLINE Search Strategy. [file 12885_2022_9261_MOESM2_ESM.docx]

**Additional file 2: Table S2. MEDLINE Search Strategy**

| **January 1, 2000 to August 31, 2020, English only  Search executed: September 30, 2020** | | |
| --- | --- | --- |
| **#** | **String** | **Hits** |
| 1 | breast neoplasms [mesh] OR “breast cancer” [tiab] OR “breast cancer” [ot] | 374,128 |
| 2 | mammography [mesh] OR mammogra* [tiab] OR mammogra* [ot] OR “breast tomosynthesis” [tiab] OR “breast tomosynthesis” [ot] | 41,183 |
| 3 | 1 AND 2 | 32,225 |
| 4 | uterine cervical neoplasms [mesh] OR “cervical cancer” [tiab] OR “cervical cancer” [ot] | 90,472 |
| 5 | Papanicolaou test [mesh] OR Papanicolaou test* [tiab] OR Papanicolaou test* [ot] OR Pap smear* [tiab] OR Pap smear* [ot] OR Pap test* [tiab] OR Pap test* [tiab] OR Pap test* [ot] OR human papillomavirus test* [tiab] OR human papillomavirus test* [ot] OR HPV test*[tiab] OR HPV test*[ot] | 14,246 |
| 6 | 4 AND 5 | 9,573 |
| 7 | colonic neoplasms [mesh] OR “colon cancer” [tiab] OR “colon cancer” [ot] | 98,923 |
| 8 | colonoscopy [mesh:noexp] OR colonoscop* [tiab] OR colonoscop* [ot] OR sigmoidoscop* [tiab] | 41,795 |
| 9 | fecal immunochemical test* [tiab] OR fecal immunochemical test* [ot] OR faecal immunochemical test* [tiab] OR faecal immunochemical test* [ot] OR FIT [tiab] OR fecal occult blood test* [tiab] OR fecal occult blood test* [ot] OR faecal occult blood test* [tiab] OR faecal occult blood test* [ot] OR FOBT [tiab] OR stool DNA test [tiab] OR stool DNA test [ot] OR cologuard [tiab] | 129,801 |
| 10 | 7 AND (8 OR 9) | 7,246 |
| 11 | lung neoplasms [mesh] OR “lung cancer” [tiab] OR “lung cancer” [ot] | 283,766 |
| 12 | (“low dose” [tiab] AND (tomography, x-ray computed [mesh] OR tomography, spiral computed [mesh])) OR “low-dose computed tomography” [tiab] OR “low-dose computed tomography” [ot] OR LDCT [tiab] | 5,575 |
| 13 | 11 AND 12 | 1,813 |
| 14 | prostatic neoplasms [mesh] OR “prostate cancer”[tiab] OR “prostate cancer”[ot] | 159,154 |
| 15 | prostate-specific antigen test* [tiab] OR prostate-specific antigen test* [ot] OR PSA test* [tiab] OR PSA test* [ot] | 2,598 |
| 16 | 14 AND 15 | 2,379 |
| 17 | ovarian neoplasms [mesh] OR “ovarian cancer” [tiab] OR “ovarian cancer” [ot] | 100,205 |
| 18 | “transvaginal ultrasound” [tiab] OR “transvaginal ultrasound” [ot] OR TVUS [tiab] | 3,971 |
| 19 | 17 AND 18 | 344 |
| 20 | early detection of cancer [mesh] OR cancer screening test* [tiab] OR “cancer screening” [tiab] | 47,342 |
| 21 | 3 OR 6 OR 10 OR 13 OR 16 OR 19 OR 20 | 89,590 |
| 22 | diagnosis [mesh:noexp] OR diagnosi* [tiab] OR diagnosis [ot] OR diagnoses [tiab] | 1,754,197 |
| 23 | 21 NOT 22 | 67,571 |
| 24 | patient reported outcome measures [mesh] OR patient reported outcome* [tiab] OR patient reported outcome* [ot] | 25,104 |
| 25 | quality of life [mesh] OR “quality of life” [tiab] OR “quality of life” [ot] OR “QoL” [tiab] OR ”quality of well-being” [tiab] OR ”quality of wellbeing” [tiab] OR ”quality of well being” [tiab] | 353,891 |
| 26 | survey* [tiab] OR questionnaire* [tiab] OR "EQ 5D" [tiab] OR "EQ5D" [tiab] OR "EuroQol" [tiab] OR "Euro Qol" [tiab] OR "SF-12" [tiab] OR "SF6D" [tiab] OR "SF 6D" [tiab] OR "short form" [tiab] OR "short forms" [tiab] | 1,124,241 |
| 27 | 24 OR 25 OR 26 | 1,376,940 |
| 28 | “health state” [tiab] OR “health state” [ot] OR “functional status” [tiab] OR “functional status” [ot] OR disabilit* [tiab] OR disabilit* [ot] OR disutility [tiab] OR disutility [ot] OR “health state utility” [tiab] OR “health state utility” [ot] OR “health state utility value” [tiab] OR “health state utility value” [ot] | 228,673 |
| 29 | patient preference [mesh] OR patient preference* [tiab] OR patient preference* [ot] OR “patient satisfaction” [tiab] OR “patient satisfaction” [ot] OR patient experience* [tiab] OR patient experience* [ot] | 73,009 |
| 30 | emotions [mesh] OR emotion* [tiab] OR emotion* [ot] OR anxiety [mesh] OR anxiety [tiab] OR anxiety [ot] OR depression [mesh] OR depress* [tiab] OR depress* [ot] OR fear [mesh] OR fear [tiab] OR fear [ot] OR fright [tiab] OR worry [tiab] OR worry [ot] OR worried [tiab] OR stress, psychological [mesh] OR psychological stress [tiab] OR psychological stress [ot] OR psychological distress [mesh] OR psychological distress [tiab] OR psychological distress [ot] | 1,020,632 |
| 31 | false positive reactions [mesh] OR false positive* [tiab] OR false-positive* [tiab] OR diagnostic uncertaint* [tiab] OR diagnostic uncertaint* [ot] | 77,978 |
| 32 | 28 OR 29 OR 30 OR 31 | 1,357,891 |
| 33 | 27 AND 32 | 282,915 |
| 34 | 23 AND 33 | 1,411 |
| 35 | valid* [tiab] OR valid* [ot] OR reliability [tiab] OR reliability [ot] | 893,445 |
| 36 | general surgery [mesh] OR surgery [tiab] OR surgical [tiab] OR anesthesia [mesh] OR anesthetics [mesh] OR sedation [tiab] OR therapeutics [mesh] OR treatment [tiab] | 8,977,424 |
| 37 | survivors [mesh] OR survivor* [ti] | 45,676 |
| 38 | knowledge [mesh:noexp] OR knowledge [tiab] OR belief [tiab] OR belief [ot] OR believ* [tiab] OR believ* [ot] | 986,047 |
| 39 | disparit* [tiab] OR disparit* [ot] OR adhere* [tiab] OR adhere* [ot] | 268,682 |
| 40 | crohn disease [mesh] OR “crohn’s disease” [tiab] OR “crohns disease” [tiab] OR ulcerative colitis [mesh] OR “ulcerative colitis” [tiab] OR irritable bowel syndrome [mesh] OR irritable bowel syndrome [tiab] OR IBS [tiab] OR IBS [ot] | 101,628 |
| 41 | health belief model* [tiab] OR health belief model* [ot] OR health literacy [mesh] OR “health literacy” [tiab] OR patient education [mesh] OR “patient education” [tiab] OR barrier* [tiab] OR barrier* [ot] | 422,806 |
| 42 | cost-benefit analysis [mesh] OR cost-effectiveness [tiab] | 114,452 |
| 43 | genetic* [tiab] OR genetic* [ot] OR mutation* [tiab] | 1,554,808 |
| 44 | 35 OR 36 OR 37 OR 38 OR 39 OR 40 OR 41 OR 42 OR 43 | 11,868,488 |
| 45 | 34 NOT 44 | 409 |
| 46 | Limit 45 to 2000/01/01 – 2020/08/31 | 348 |
| 47 | Limit 46 to English | 338 |
