# Supplementary material for: Patient-reported outcomes associated with cancer screening: a systematic review
Source: BMC Cancer. 2022 Mar 1;22:223. doi: 10.1186/s12885-022-09261-5 (PMC8886782; doi:10.1186/s12885-022-09261-5)
Supplement: Supplementary file 3 — Additional file 3: Table S3. EMBASE Search Strategy. [file 12885_2022_9261_MOESM3_ESM.docx]

**Additional file 3: Table S3. EMBASE Search Strategy**

| **January 2000 to August 2020, English only  Search executed: October 15, 2020** | | |
| --- | --- | --- |
| **#** | **String** | **Hits** |
| 1 | breast neoplasms'/exp OR 'breast cancer':ab,ti,kw | 608,888 |
| 2 | mammography'/exp OR 'mammogra*':ab,ti,kw OR 'breast tomosynthesis':ab,ti,kw | 65,411 |
| 3 | #1 AND #2 | 50,796 |
| 4 | uterine cervical neoplasms'/exp OR 'cervical cancer':ab,ti,kw | 133,427 |
| 5 | papanicolaou test'/exp OR 'papanicolaou test*':ab,ti,kw OR 'pap smear*':ab,ti,kw OR 'pap test*':ab,ti,kw OR 'human papillomavirus test*':ab,ti,kw OR 'hpv test*':ab,ti,kw | 25,471 |
| 6 | #4 AND #5 | 15,340 |
| 7 | colonic neoplasms'/exp OR 'colon cancer':ab,ti,kw | 348,737 |
| 8 | colonoscopy'/de OR 'colonoscop*':ab,ti,kw OR 'sigmoidoscop*':ab,ti | 90,502 |
| 9 | fecal immunochemical test*':ab,ti,kw OR 'faecal immunochemical test*':ab,ti,kw OR 'fit':ab,ti OR 'fecal occult blood test*':ab,ti,kw OR 'faecal occult blood test*':ab,ti,kw OR 'fobt':ab,ti OR 'stool dna test*':ab,ti,kw OR 'cologuard':ab,ti | 165,072 |
| 10 | #7 AND (#8 OR #9) | 40,089 |
| 11 | lung neoplasms'/exp OR 'lung cancer':ab,ti,kw | 472,498 |
| 12 | low dose':ab,ti AND ('tomography, x-ray computed'/exp OR 'tomography, spiral computed'/exp) OR 'low-dose computed tomography':ab,ti,kw OR 'ldct':ab,ti | 4,052 |
| 13 | #11 AND #12 | 2,315 |
| 14 | prostatic neoplasms'/exp OR 'prostate cancer':ab,ti,kw | 267,446 |
| 15 | prostate-specific antigen test':ab,ti,kw OR 'psa test*':ab,ti,kw | 3,619 |
| 16 | #14 AND #15 | 3,303 |
| 17 | ovarian neoplasms'/exp OR 'ovarian cancer':ab,ti,kw | 162,231 |
| 18 | transvaginal ultrasound':ab,ti,kw OR 'tvus':ab,ti | 6,826 |
| 19 | #17 AND #18 | 612 |
| 20 | cancer screening tests' OR 'cancer screening test*':ab,ti OR 'cancer screening':ab,ti | 43,905 |
| 21 | #3 OR #6 OR #10 OR #13 OR #16 OR #19 OR #20 | 134,695 |
| 22 | diagnosis'/de OR 'diagnosi*':ab,ti,kw OR 'diagnoses':ab,ti | 3,223,155 |
| 23 | #21 NOT #22 | 97,242 |
| 24 | patient reported outcome measures'/exp OR 'patient reported outcome*':ab,ti,kw | 42,532 |
| 25 | quality of life'/exp OR 'quality of life':ab,ti,kw OR 'qol':ab,ti OR 'quality of well-being':ab,ti OR 'quality of wellbeing':ab,ti OR 'quality of well being':ab,ti | 608,589 |
| 26 | questionnaire*':ab,ti OR 'survey':ab,ti OR 'eq5d':ab,ti OR 'eq 5d':ab,ti OR 'euroqol':ab,ti OR 'euro qol':ab,ti OR 'sf-12':ab,ti OR 'sf12':ab,ti OR 'sf6d':ab,ti OR 'sf 6d':ab,ti OR 'short form':ab,ti OR 'short forms':ab,ti | 1,335,047 |
| 27 | #24 OR #25 OR #26 | 1,792,046 |
| 28 | health state':ab,ti,kw OR 'functional status':ab,ti,kw OR 'disabilit*':ab,ti,kw OR 'disutility':ab,ti,kw OR 'health state utility':ab,ti,kw OR 'health state utility value':ab,ti,kw | 318,429 |
| 29 | patient preference'/exp OR 'patient preference*':ab,ti,kw OR 'patient satisfaction':ab,ti,kw OR 'patient experience*':ab,ti,kw | 107,836 |
| 30 | emotions'/exp OR 'emotion*':ab,ti,kw OR 'anxiety'/exp OR 'anxiety':ab,ti,kw OR 'depression'/exp OR 'depress*':ab,ti,kw OR 'fear'/exp OR 'fear':ab,ti,kw OR 'fright':ab,ti OR 'worry':ab,ti,kw OR 'worried':ab,ti OR 'stress, psychological'/exp OR 'psychological stress':ab,ti,kw OR 'psychological distress'/exp OR 'psychological distress':ab,ti,kw | 1,521,220 |
| 31 | false positive reactions'/exp OR 'false positive*':ab,ti OR 'false-positive*':ab,ti OR 'diagnostic uncertaint*':ab,ti,kw | 95,669 |
| 32 | #28 OR #29 OR #30 OR #31 | 1,971,165 |
| 33 | #27 AND #32 | 419,138 |
| 34 | #23 AND #33 | 2,029 |
| 35 | genetic*':ab,ti,kw OR 'mutation*':ab,ti | 1,918,555 |
| 36 | valid*':ab,ti,kw OR 'reliability':ab,ti,kw | 1,182,812 |
| 37 | general surgery'/exp OR 'surgery':ab,ti OR 'surgical':ab,ti OR 'anesthesia'/exp OR 'anesthetics'/exp OR 'sedation':ab,ti OR 'therapeutics'/exp OR 'treatment*':ab,ti | 13,709,450 |
| 38 | survivors'/exp OR 'survivor*':ab,ti | 156,455 |
| 39 | knowledge'/de OR 'knowledge':ab,ti OR 'belief':ab,ti,kw OR 'believ*':ab,ti,kw | 1,206,460 |
| 40 | disparit*':ab,ti,kw OR 'adhere*':ab,ti,kw | 364,485 |
| 41 | crohn disease'/exp OR 'crohn/s disease':ab,ti OR 'crohns disease':ab,ti OR 'ulcerative colitis'/exp OR 'ulcerative colitis':ab,ti OR 'irritable bowel syndrome'/exp OR 'irritable bowel syndrome':ab,ti,kw OR 'ibs':ab,ti,kw | 176,468 |
| 42 | health belief model*':ab,ti,kw OR 'health literacy'/exp OR 'health literacy':ab,ti OR 'patient education'/exp OR 'patient education':ab,ti OR 'barrier*':ab,ti,kw | 508,402 |
| 43 | cost-benefit analysis'/exp OR 'cost-effectiveness':ab,ti | 159,262 |
| 44 | #35 OR #36 OR #37 OR #38 OR #39 OR #40 OR #41 OR #42 OR #43 | 16,793,951 |
| 45 | #34 NOT #44 | 426 |
| 46 | #45 AND [1-1-2000]/sd NOT [31-8-2020]/sd | 374 |
| 47 | #46 AND [english]/lim | 362 |
| 48 | #47 AND [embase]/lim | 297 |
