# Supplementary material for: Patient-reported outcomes associated with cancer screening: a systematic review
Source: BMC Cancer. 2022 Mar 1;22:223. doi: 10.1186/s12885-022-09261-5 (PMC8886782; doi:10.1186/s12885-022-09261-5)
Supplement: Supplementary file 7 — Additional file 7: Table S7. Patient-Reported Outcomes Related to Functional Status and Well-Being. [file 12885_2022_9261_MOESM7_ESM.docx]

**Additional file 7: Table S7. Patient-Reported Outcomes Related to Functional Status and Well-Being**

|  |  |  |  |  | |  | **Screening** | |  | **2 mo** | | **3 mo** | | | **4 mo** | | **5 mo** | | **6 mo** | | **7 mo - 11 mo** | | | | | | **12 mo** | |
| --- | --- | --- | --- | --- | --- | --- | --- | --- | --- | --- | --- | --- | --- | --- | --- | --- | --- | --- | --- | --- | --- | --- | --- | --- | --- | --- | --- | --- |
|  | **Study Design** | **Screening** | **Measure**^†^ | **Result** | | **Baseline** | | **Within 1 mo** | |  |  |  |  | |  |  |  |  |  |  | | | | | | | |  |
|  |  |  |  |  |  | **Mean (SD)** | | **Mean (SD)** | | **Mean (SD)** | | | | | | | | | | | | | | | | | | |
| **Cvejic et al 2020^67^**  N=270–617   - Age: ≥35 years - HIV (+/-) GBMSM in the SPANC | Prospective cohort | Anal Swab + HRA | SF-36 MCS | Total (Abnormal, Normal) | | 48.9 (10.4) | | AMD: 1.79  (-2.99–0.59)^a^ | |  | | AMD: 0.79  (-2.68–1.10)^b^ | | | |  | |  |  | | |  |  |  |  |  |  | |
| **Taghizadeh et al 2019^34^**  N-953–1,237 (total)  N=238–279 (+)   - Age: 50–75 years - Pan-Canadian Early Detection of Lung Cancer Study | Prospective cohort | LDCT | SF-12 MCS | Total (+/–) | | 51.1 | | 50.9 | |  | |  | | | |  | |  |  | | |  |  |  |  |  | 51.2 | |
|  |  |  |  | (+) | | 51.3 | | 51.2 | |  | |  | | | |  | |  |  | | |  |  |  |  |  | 51.3 | |
|  |  |  | SF-12 PCS | Total (+/–) | | 46.1 | | 46.8 | |  | |  | | | |  | |  |  | | |  |  |  |  |  | 46.4 | |
|  |  |  |  | (+) | | 46.2 | | 46.5 | |  | |  | | | |  | |  |  | | |  |  |  |  |  | 45.3 | |
| **Kirkoen et al 2016^36^**  N=1,523–2,803 (FS)  N=1,601–3,462 (FIT)   - Age: 50–74 years - BCSN pilot participants | Randomized screening | FS | SF-12 | (+) | PF | 82.2 | | 80.5^c^ | |  | |  | | | |  | |  | 79.8 | | |  |  |  |  |  | 79.2 | |
|  |  |  |  |  | RP | 75.9 | | 78.1^c^ | |  | |  | | | |  | |  | 76.9 | | |  |  |  |  |  | 76.4 | |
|  |  |  |  |  | RE | 81.6 | | 81.0^c^ | |  | |  | | | |  | |  | 79.1 | | |  |  |  |  |  | 79.8 | |
|  |  |  |  |  | MH | 79.3 | | 79.2^c^ | |  | |  | | | |  | |  | 80.8 | | |  |  |  |  |  | 79.4 | |
|  |  |  |  |  | GH | 64.3 | | 63.8^c^ | |  | |  | | | |  | |  | 63.5 | | |  |  |  |  |  | 62.9 | |
|  |  |  |  |  | BP | 79.6 | | 80.8^c^ | |  | |  | | | |  | |  | 78.3 | | |  |  |  |  |  | 80.8 | |
|  |  |  |  |  | VT | 57.2 | | 58.3^c^ | |  | |  | | | |  | |  | 59.5 | | |  |  |  |  |  | 57.1 | |
|  |  |  |  |  | SF | 85.3 | | 86.6^c^ | |  | |  | | | |  | |  | 87.7 | | |  |  |  |  |  | 85.4 | |
|  |  | FIT |  | (–) | PF | 86.2 | | 86.3^c^ | |  | |  | | | |  | |  | 85.1 | | |  |  |  |  |  | 84.9 | |
|  |  |  |  |  | RP | 82.4 | | 81.8^c^ | |  | |  | | | |  | |  | 80.3 | | |  |  |  |  |  | 79.2 | |
|  |  |  |  |  | RE | 87.5 | | 87.3^c^ | |  | |  | | | |  | |  | 86.6 | | |  |  |  |  |  | 85.2 | |
|  |  |  |  |  | MH | 81.3 | | 82.3^c^ | |  | |  | | | |  | |  | 81.7 | | |  |  |  |  |  | 80.3 | |
|  |  |  |  |  | GH | 69.0 | | 70.2^c^ | |  | |  | | | |  | |  | 68.8 | | |  |  |  |  |  | 68.8 | |
|  |  |  |  |  | BP | 83.0 | | 83.7^c^ | |  | |  | | | |  | |  | 82.5 | | |  |  |  |  |  | 82.2 | |
|  |  |  |  |  | VT | 60.8 | | 62.8^c^ | |  | |  | | | |  | |  | 61.6 | | |  |  |  |  |  | 59.5 | |
|  |  |  |  |  | SF | 89.5 | | 90.2^c^ | |  | |  | | | |  | |  | 89.4 | | |  |  |  |  |  | 88.3 | |
| **Kirkoen et al 2016^35^**  N=2,383–2,906 (FS)  N=1,730–3,521 (FIT)   - Age: 50–74 years - BCSN pilot participants | Randomized screening | FS | SF-12 | (+) | PF | 86.7 (22.6) | | 84.1 (22.1)^c^ | |  | |  | | | |  | |  |  | | |  |  |  |  |  |  | |
|  |  |  |  |  | RP | 84.2 (33.9) | | 84.6 (33.9)^c^ | |  | |  | | | |  | |  |  | | |  |  |  |  |  |  | |
|  |  |  |  |  | RE | 81.7 (27.2) | | 85.7 (28.1)^c^ | |  | |  | | | |  | |  |  | | |  |  |  |  |  |  | |
|  |  |  |  |  | MH | 83.4 (17.4) | | 81.7 (17.5)^c^ | |  | |  | | | |  | |  |  | | |  |  |  |  |  |  | |
|  |  |  |  |  | GH | 69.4 (21.3) | | 69.5 (21.3)^c^ | |  | |  | | | |  | |  |  | | |  |  |  |  |  |  | |
|  |  |  |  |  | BP | 82.9 (21.4) | | 85.4 (21.6)^c^ | |  | |  | | | |  | |  |  | | |  |  |  |  |  |  | |
|  |  |  |  |  | VT | 62.5 (24.2) | | 62.9 (24.2)^c^ | |  | |  | | | |  | |  |  | | |  |  |  |  |  |  | |
|  |  |  |  |  | SF | 87.8 (18.4) | | 87.9 (17.7)^c^ | |  | |  | | | |  | |  |  | | |  |  |  |  |  |  | |
|  |  |  |  | (–) | PF | 87.0 (23.1) | | 87.4 (25.9)^c^ | |  | |  | | | |  | |  |  | | |  |  |  |  |  |  | |
|  |  |  |  |  | RP | 84.5 (31.9) | | 83.9 (31.9)^c^ | |  | |  | | | |  | |  |  | | |  |  |  |  |  |  | |
|  |  |  |  |  | RE | 90.2 (27.9) | | 89.9 (27.9)^c^ | |  | |  | | | |  | |  |  | | |  |  |  |  |  |  | |
|  |  |  |  |  | MH | 83.6 (16.0) | | 84.3 (16.0)^c^ | |  | |  | | | |  | |  |  | | |  |  |  |  |  |  | |
|  |  |  |  |  | GH | 69.5 (20.0) | | 72.1 (20.0)^c,^* | |  | |  | | | |  | |  |  | | |  |  |  |  |  |  | |
|  |  |  |  |  | BP | 84.5 (25.1) | | 85.7 (22.4)^c,^* | |  | |  | | | |  | |  |  | | |  |  |  |  |  |  | |
|  |  |  |  |  | VT | 62.9 (25.1) | | 65.3 (24.7)^c,^* | |  | |  | | | |  | |  |  | | |  |  |  |  |  |  | |
|  |  |  |  |  | SF | 91.5 (18.8) | | 92.2 (18.0)^c^ | |  | |  | | | |  | |  |  | | |  |  |  |  |  |  | |
|  |  | FIT |  | (+) | PF | 79.5 (27.2) | | 80.1 (26.6)^c^ | |  | |  | | | |  | |  |  | | |  |  |  |  |  |  | |
|  |  |  |  |  | RP | 73.8 (39.7) | | 74.6 (40.7)^c^ | |  | |  | | | |  | |  |  | | |  |  |  |  |  |  | |
|  |  |  |  |  | RE | 85.1 (32.9) | | 77.2 (33.9)^c^ | |  | |  | | | |  | |  |  | | |  |  |  |  |  |  | |
|  |  |  |  |  | MH | 79.4 (20.4) | | 78.9 (20.4)^c^ | |  | |  | | | |  | |  |  | | |  |  |  |  |  |  | |
|  |  |  |  |  | GH | 66.4 (25.2) | | 64.2 (25.2)^c^ | |  | |  | | | |  | |  |  | | |  |  |  |  |  |  | |
|  |  |  |  |  | BP | 79.3 (25.5) | | 80.8 (26.0)^c^ | |  | |  | | | |  | |  |  | | |  |  |  |  |  |  | |
|  |  |  |  |  | VT | 58.9 (29.9) | | 60.4 (29.5)^c^ | |  | |  | | | |  | |  |  | | |  |  |  |  |  |  | |
|  |  |  |  |  | SF | 84.6 (21.6) | | 89.1 (20.9)^c^ | |  | |  | | | |  | |  |  | | |  |  |  |  |  |  | |
|  |  |  |  | (–) | PF | 85.8 (26.7) | | 85.2 (26.3)^c^ | |  | |  | | | |  | |  |  | | |  |  |  |  |  |  | |
|  |  |  |  |  | RP | 82.0 (35.9) | | 80.8 (39.9)^c^ | |  | |  | | | |  | |  |  | | |  |  |  |  |  |  | |
|  |  |  |  |  | RE | 87.8 (31.9) | | 86.6 (31.9)^c^ | |  | |  | | | |  | |  |  | | |  |  |  |  |  |  | |
|  |  |  |  |  | MH | 80.8 (19.5) | | 81.6 (20.0)^c^ | |  | |  | | | |  | |  |  | | |  |  |  |  |  |  | |
|  |  |  |  |  | GH | 69.8 (23.9) | | 69.7 (23.9)^c^ | |  | |  | | | |  | |  |  | | |  |  |  |  |  |  | |
|  |  |  |  |  | BP | 83.5 (25.5) | | 83.1 (25.9)^c^ | |  | |  | | | |  | |  |  | | |  |  |  |  |  |  | |
|  |  |  |  |  | VT | 61.7 (29.1) | | 63.5 (28.7)^c,^* | |  | |  | | | |  | |  |  | | |  |  |  |  |  |  | |
|  |  |  |  |  | SF | 88.8 (21.6) | | 89.2 (21.2)^c^ | |  | |  | | | |  | |  |  | | |  |  |  |  |  |  | |
| **Ong et al 2016^50^**  N=234–327   - Age: ≥35+ years - HIV (+) MSM | Prospective questionnaire | DARE | SF-12 | Total (Abnormal, Normal) | | NR | | NR | |  | |  | | | |  | |  |  | | |  |  |  |  |  |  | |
| **Gareen et al 2014^71^**  N=1,990–2,812   - Age: 55–74 years - NLST participants | Randomized screening | LDCT | SF-36v2 MCS | TP | | 52.0 (11.0) | | 44.1 (14.0)*^,g^ | |  | |  | | | |  | |  | 46.3 (13.7)*^,g^ | | |  |  |  |  |  |  | |
|  |  |  |  | SIF | | 51.5 (9.8) | | 50.2 (11.9) | |  | |  | | | |  | |  | 57.8 (10.5) | | |  |  |  |  |  |  | |
|  |  |  |  | FP | | 57.8 (10.0) | | 50.6 (10.7) | |  | |  | | | |  | |  | 50.4 (11.2) | | |  |  |  |  |  |  | |
|  |  |  |  | (–) | | 51.9 (10.2) | | 51.3 (10.6) | |  | |  | | | |  | |  | 51.4 (10.6) | | |  |  |  |  |  |  | |
|  |  | CXR |  | TP | | 53.8 (8.6) | | 51.4 (9.9)*^,g^ | |  | |  | | | |  | |  | 46.2 (12.2)*^,g^ | | |  |  |  |  |  |  | |
|  |  |  |  | SIF | | 50.9 (10.1) | | 50.0 (10.9) | |  | |  | | | |  | |  | 51.0 (9.2) | | |  |  |  |  |  |  | |
|  |  |  |  | FP | | 58.0 (9.2) | | 51.1 (10.4) | |  | |  | | | |  | |  | 51.4 (9.8) | | |  |  |  |  |  |  | |
|  |  |  |  | (–) | | 52.7 (9.7) | | 51.1 (11.0) | |  | |  | | | |  | |  | 51.5 (10.4) | | |  |  |  |  |  |  | |
|  |  | LDCT | SF-36v2 PCS | TP | | 46.6 (11.3) | | 44.5 (11.6) | |  | |  | | | |  | |  | 38.3 (12.6) | | |  |  |  |  |  |  | |
|  |  |  |  | SIF | | 48.4 (9.3) | | 47.9 (9.4) | |  | |  | | | |  | |  | 47.2 (9.4) | | |  |  |  |  |  |  | |
|  |  |  |  | FP | | 47.9 (10.0) | | 47.7 (10.2) | |  | |  | | | |  | |  | 47.1 (10.2) | | |  |  |  |  |  |  | |
|  |  |  |  | (–) | | 48.2 (9.0) | | 47.6 (9.5) | |  | |  | | | |  | |  | 48.0 (9.2) | | |  |  |  |  |  |  | |
|  |  | CXR |  | TP | | 48.5 (9.9) | | 42.1 (11.1) | |  | |  | | | |  | |  | 38.5 (10.0) | | |  |  |  |  |  |  | |
|  |  |  |  | SIF | | 48.2 (10.5) | | 47.6 (10.1) | |  | |  | | | |  | |  | 48.5 (9.6) | | |  |  |  |  |  |  | |
|  |  |  |  | FP | | 48.6 (9.5) | | 48.6 (9.5) | |  | |  | | | |  | |  | 47.9 (10.2) | | |  |  |  |  |  |  | |
|  |  |  |  | (–) | | 49.2 (9.0) | | 48.9 (9.6) | |  | |  | | | |  | |  | 48.0 (9.9) | | |  |  |  |  |  |  | |
| **Vasarainen et al 2013^70^**  N=271–386   - Age: 50–74 years - ERSPC participants | Randomized screening | PSA | SF-36 | PF | | 90 (80–95) | | 90 (90–100)^d^ | | 90 (80–95)^c^ | | | |  | |  | |  |  | | |  |  |  |  |  |  | |
|  |  |  |  | RP | | 100 (75–100) | | 100 (75–100)^d^ | | 100 (75–100)^c^ | | | |  | |  | |  |  | | |  |  |  |  |  |  | |
|  |  |  |  | RE | | 100 (67–100) | | 100 (67–100)^d^ | | 100 (67–100)^c^ | | | |  | |  | |  |  | | |  |  |  |  |  |  | |
|  |  |  |  | MH | | 84 (69–92) | | 84 (72–92)^d^ | | 80 (72–92)^c^ | | | |  | |  | |  |  | | |  |  |  |  |  |  | |
|  |  |  |  | GH | | 65 (50–75) | | 65 (50–75)^d^ | | 65 (50–75)^c^ | | | |  | |  | |  |  | | |  |  |  |  |  |  | |
|  |  |  |  | BP | | 90 (68–100) | | 90 (68–100)^d^ | | 90 (68–100)^c^ | | | |  | |  | |  |  | | |  |  |  |  |  |  | |
|  |  |  |  | VT | | 70 (55–85) | | 75 (60–85)^d^ | | 75 (60–85)^c^ | | | |  | |  | |  |  | | |  |  |  |  |  |  | |
|  |  |  |  | SF | | 100 (75–100) | | 100 (75–100)^d^ | | 95 (75–100)^c,^* | | | |  | |  | |  |  | | |  |  |  |  |  |  | |
| **Landstra et al 2013^51^**  N=50–271   - HIV+ and MSM with history of (non-cancer) anal disease | Prospective longitudinal survey | Anal Swab + HRA | SF-12 | Total (+/–/FP) | | NR | | NR | |  | | | |  | | NR | |  |  | | |  |  |  |  |  |  | |
| **Korfage et al 2012^17^**  N=706–789   - Age: 30–60 years - Women living in Netherlands | Prospective cohort questionnaire | Pap | SF-12 MCS | Normal | | 53 (9) | | 53 (9)^d^ | |  | | | |  | |  | |  |  | | |  |  |  |  |  |  | |
|  |  |  | SF-12 PCS |  |  | 52 (9) | | 52 (9)^d^ | |  | | | |  | |  | |  |  | | |  |  |  |  |  |  | |
| **Hafslund et al 2012^39^**  N=77–128 (FP)   - Age: 50-69 years | Prospective descriptive longitudinal | MMG | SF-36 | FP | MH | 83.0 (14.5) | | 79.9 (16.5)* | |  | | | | 81.5 (13.5) | | | |  |  | | | 83.0 (14.5)^XX^ | |  |  |  |  | |
|  |  |  |  |  | GH | 74.6 (20.7) | | 72.6 (21.9) | |  | | | | 71.0 (22.9) | | | |  |  | | | 70.3 (23.7)^XX^ | |  |  |  |  | |
|  |  |  |  | (–) | MH | 83.0 (14.7) | | NA | |  | | | | NA | | | |  |  | | | 83.0 (14.7) | |  |  |  |  | |
|  |  |  |  |  | GH | 77.0 (19.8) | | NA | |  | | | | NA | | | |  |  | | | 77.0 (20.0) | |  |  |  |  | |
| **van den Bergh et al 2011^48^**  N=600–658   - Age: 50–75 years - NELSON study participants | RCT | LDCT | SF-12 MCS | Indeterminate | | 52.0 (50.1–53.4) | |  | | 52.0 (50.0–54.0) | | | |  | | | |  |  | | |  | |  |  |  |  | |
|  |  |  |  | (–) | | 51.9 (50.8–52.9) | |  | | 51.5 (50.5–52.5) | | | |  | | | |  |  | | |  | |  |  |  |  | |
|  |  |  | SF-12 PCS | Indeterminate | | 48.7 (47.1–50.2) | |  | | 49.3 (47.7–50.9) | | | |  | | | |  |  | | |  | |  |  |  |  | |
|  |  |  |  | (–) | | 49.9 (49.1–50.7) | |  | | 50.4 (49.6–51.3) | | | |  | | | |  |  | | |  | |  |  |  |  | |
| **van den Bergh et al 2010^41^**  N=600–630   - Age: 50–75 years - NELSON study participants | RCT | LDCT | SF-12 MCS | Indeterminate | | 51.8 (10.6) | |  | | 51.9 (11.0) | | | |  | | | |  |  | | |  | |  |  |  |  | |
|  |  |  |  | (–) | | 51.9 (10.2) | |  | | 51.6 (11.1) | | | |  | | | |  |  | | |  | |  |  |  |  | |
|  |  |  | SF-12 PCS | Indeterminate | | 48.5 (9.6) | |  | | 48.9 (7.8) | | | |  | | | |  |  | | |  | |  |  |  |  | |
|  |  |  |  | (–) | | 49.7 (8.4) | |  | | 50.3 (8.3) | | | |  | | | |  |  | | |  | |  |  |  |  | |
| **van den Bergh et al 2008^43^**  N=268–324   - Age: 50–75 years - NELSON study participants | RCT | LDCT | SF-12 MCS | Total  (–/Incidental) | | 54.2^h^ | |  | |  | | | |  | | | |  |  | | | 55.4^h^ | |  |  |  |  | |
|  |  |  | SF-12 PCS |  |  | 51.3^h^ | |  | |  | | | |  | | | |  |  | | | 51.9^h^ | |  |  |  |  | |
| **Bunge et al 2008^49^**  N=40–47 (High AR)  N=236–274 (Low AR)   - Age: 50–75 years - NELSON study participants | RCT | LDCT | SF-12 MCS | High AR | | 50.4^e,h^ | | High AR | |  | | | |  | | | |  | 52.2^h^ | | |  | |  |  |  |  | |
|  |  |  |  | Low AR | | 54.5^h^ | | Low AR | |  | | | |  | | | |  | 55.5^h^ | | |  | |  |  |  |  | |
|  |  |  | SF-12 MCS | High AR | | 51.9^h^ | | High AR | |  | | | |  | | | |  | 52.0^h^ | | |  | |  |  |  |  | |
|  |  |  |  | Low AR | | 51.2^h^ | | Low AR | |  | | | |  | | | |  | 52.0^h^ | | |  | |  |  |  |  | |
| **Wood et al 2008^42^**  N=15   - Age: ≥25 years - Referred women from HNPCC or Lynch syndrome families | Observational | OPH + EB + TVS + CA125 | SF-36 MCS | Total (–/FP) | | 52.9 (9.3) | |  | | 53.1 (11.3) | | | |  | | | |  | 50.8 (10.2) | | |  | |  |  |  |  | |
|  |  |  |  | FP | | 57.0 (53–61.1) | |  | | 55.0 (46.3–63.7) | | | |  | | | |  | 53.6 (45.4–61.8) | | |  | |  |  |  |  | |
|  |  |  | SF-36 PCS | Total (–/FP) | | 50.8 (9.3) | |  | | 46.6 (13.8) | | | |  | | | |  | 47.3 (15.0) | | |  | |  |  |  |  | |
|  |  |  |  | FP | | 53.0 (48.6–57.7) | |  | | 45.8 (34.3–57.3) | | | |  | | | |  | 46.2 (32.7–59.8) | | |  | |  |  |  |  | |
| **Taupin et al 2006^69^**  N=231   - 55–74 years - Asymptomatic average-risk - Colonoscopy within 10 years or cancer history | Population-based screening | Colonoscopy | SF-36 | PF | | 80.2 | | 78.6^f^ | |  | | | |  | | | |  |  | | |  | |  |  |  |  | |
|  |  |  |  | RP | | 82.6 | | 85.2^f^ | |  | | | |  | | | |  |  | | |  | |  |  |  |  | |
|  |  |  |  | RE | | 88.6 | | 92.9^f,^* | |  | | | |  | | | |  |  | | |  | |  |  |  |  | |
|  |  |  |  | MH | | 78.0 | | 79.9^f,i^ | |  | | | |  | | | |  |  | | |  | |  |  |  |  | |
|  |  |  |  | GH | | 73.3 | | 72.7^f^ | |  | | | |  | | | |  |  | | |  | |  |  |  |  | |
|  |  |  |  | BP | | 75.6 | | 77.3^f^ | |  | | | |  | | | |  |  | | |  | |  |  |  |  | |
|  |  |  |  | VT | | 64.6 | | 66.5*^f,i^ | |  | | | |  | | | |  |  | | |  | |  |  |  |  | |
|  |  |  |  | SF | | 88.1 | | 88.5^f^ | |  | | | |  | | | |  |  | | |  | |  |  |  |  | |
| **Taylor et al 2004^61^**  N=163–325   - Age: 55–74 years - PLCO cancer screening trial participants | Randomized screening | DRE + PSA; CXR; FS;  TVS + CA125 | SF-12 MCS | Screen | | 54.2 (7.3) | | NA | |  | | | |  | | | |  |  | | |  | |  |  |  | NA | |
|  |  |  |  | Normal | |  |  | 52.3 (8.6) | |  | | | |  | | | |  |  | | |  | |  |  |  | 52.5 (9.7) | |
|  |  |  |  | Abnormal | |  |  | 53.9 (7.8) | |  | | | |  | | | |  |  | | |  | |  |  |  | 55.1 (5.7) | |
|  |  |  |  | Control | | 55.7 (5.1) | | NA | |  | | | |  | | | |  |  | | |  | |  |  |  | 54.4 (7.1) | |
|  |  |  | SF-12 PCS | Screen | | 52.3 (7.1) | | NA | |  | | | |  | | | |  |  | | |  | |  |  |  | NA | |
|  |  |  |  | Normal | |  |  | 53.7 (5.5) | |  | | | |  | | | |  |  | | |  | |  |  |  | 50.3 (8.5) | |
|  |  |  |  | Abnormal | |  |  | 51.1 (9.1) | |  | | | |  | | | |  |  | | |  | |  |  |  | 48.7 (10.1) | |
|  |  |  |  | Control | | 51.8 (7.5) | | NA | |  | | | |  | | | |  |  | | |  | |  |  |  | 49.1 (8.8) | |
| **Parker et al 2002^68^**  N=1,303–1,541   - Age: 45–74 years | Randomized screening | FOBT | GHQ | Total (+/–/FP) | | 29.5%  (GHQ>5) | |  | |  | | | | 29.6%^c^  (GHQ>5) | | | |  |  | | |  | |  |  |  |  | |
| **Cormier et al 2002^46^**  N=220   - Age: 40–70 years - Family history of prostate cancer | Prospective questionnaire | PSA | SF-36^j^ | PF | | 89 (19) | | 89 (19)^d^ | | 90 (18)^c^ | | | |  | | | |  |  | | |  | |  |  |  |  | |
|  |  |  |  | RP | | 88 (27) | | 85 (31)^d^ | | 87 (28)^c^ | | | |  | | | |  |  | | |  | |  |  |  |  | |
|  |  |  |  | RE | | 87 (28) | | 85 (31)^d,YY^ | | 90 (25)^c^ | | | |  | | | |  |  | | |  | |  |  |  |  | |
|  |  |  |  | MH | | 73 (18) | | 74 (17)^d,YY^ | | 76 (15)^c^ | | | |  | | | |  |  | | |  | |  |  |  |  | |
|  |  |  |  | GH | | 69 (18) | | 70 (18)^d^ | | 71 (17)^c^ | | | |  | | | |  |  | | |  | |  |  |  |  | |
|  |  |  |  | BP | | 74 (23) | | 73 (23)^d^ | | 74 (22)^c^ | | | |  | | | |  |  | | |  | |  |  |  |  | |
|  |  |  |  | VT | | 65 (18) | | 67 (18)^d,YY^ | | 68 (17)^c^ | | | |  | | | |  |  | | |  | |  |  |  |  | |
|  |  |  |  | SF | | 85 (12) | | 84 (22)^d^ | | 86 (19)^c^ | | | |  | | | |  |  | | |  | |  |  |  |  | |

*Indicating statistical significance, *p*<0.05.

^†^SF-12, SF-36: higher scores indicates better functional status and well-being; GHQ>5 indicates psychiatric morbidity and worse functional status and well-being.

^a^Lower scores (p=0.004) for those who perceive results as abnormal, compared to those who perceived their results as normal.

^b^Lower scores for those who perceive results as abnormal, compared to those who perceived their results as normal.

^c^Post-screening results, with no specific time point.

^d^Post-screen, with no specific time point.

^e^*p*<0.01 versus low AR group.

^f^Post-screening results, mean 36 days.

^g^Clinically important (e.g., a 3- to 5-point change in either of the SF-36 components) in those with positive results.

^h^Median values.

^I^Increase in the number of participants (30%) with clinically improved VT and MH scores post-screen.

^j^Minimally clinically important (e.g., at least 1 standard error of measurement change) decrease in functional status and well-being in approximately 20% of individuals.

^XX^Indicating statistical significance, *p*<0.05, compared to (-) group (not baseline).

^YY^Indicating statistical significance, *p*<0.05, from baseline to waiting for results and receiving normal results.

Abbreviations: AMD, adjusted mean difference; AR, affective risk; BCSN, Bowel Cancer Screening in Norway; BP, bodily pain; CA-125, ovarian tumor marker; CXR, chest x-ray; DARE, digital anal rectal examination; DRE, digital rectal exam; EB, endometrial biopsy; ERSPC, European Randomized Study of Screening for Prostate Cancer; FIT, fecal immunochemical test; FOBT, fecal occult blood test; FP, false positive; FS, flexible sigmoidoscopy; GBMSM, gay, bisexual, and other men who have sex with men; GH, general health; GHQ, general health questionnaire; HNPCC, hereditary nonpolyposis colorectal cancer; HRA, high resolution anoscopy; LDCT, low-dose computed tomography; MCS, mental component summary; MH, mental health; MMG, mammogram; MSM, men who have sex with men; NELSON, The Dutch-Belgian Randomized Lung Cancer Screening Trial; NA, not applicable; NLST, National Lung Screening Trial; NR, not reported; OPH, outpatient hysteroscopy; PCS, physical component summary; PF, physical functioning; PLCO, prostate, lung, colorectal, and ovarian; PSA, prostate-specific antigen; RCT, randomized controlled trial; RE, Role-Emotional; RP, Role-Physical; SD, standard deviation; SF, social functioning; SF-12, 12-item Short Form; SF-36, 36-item Short Form; SPANC, Study of the Prevention of Anal Cancer; TP, true positive; TVS, transvaginal ultrasound scan; VT, vitality.
