# Supplementary material for: Patient-reported outcomes associated with cancer screening: a systematic review
Source: BMC Cancer. 2022 Mar 1;22:223. doi: 10.1186/s12885-022-09261-5 (PMC8886782; doi:10.1186/s12885-022-09261-5)
Supplement: Supplementary file 8 — Additional file 8: Table S8. Patient-Reported Outcomes Related to Preference-Weighted Health Status. [file 12885_2022_9261_MOESM8_ESM.docx]

**Additional file 8: Table S8. Patient-Reported Outcomes Related to Preference-Weighted Health Status**

|  | **Study Design** | **Screening** | **Measure**^†^ | **Result** |  | **Screening** | |  | **2 mo** | | **3 mo** | | | **4 mo** | | **5 mo** | | **6 mo** | | **7 mo–11 mo** | | | | | | **12 mo** | |
| --- | --- | --- | --- | --- | --- | --- | --- | --- | --- | --- | --- | --- | --- | --- | --- | --- | --- | --- | --- | --- | --- | --- | --- | --- | --- | --- | --- |
|  |  |  |  |  | **Baseline** | | **Within 1 mo** | |  |  |  |  | |  |  |  |  |  |  | | | | | | | |  |
|  |  |  |  |  | **Mean (SD)** | | **Mean (SD)** | | **Mean (SD)** | | | | | | | | | | | | | | | | | | |
| **Cvejic et al 2020^67^**  N=270–617   - Age: ≥35 years - HIV (+/–) GBMSM in the SPANC | Prospective cohort | Anal Swab + HRA | SF-6D | Total (Abnormal, Normal) | 0.8 (0.1) | | AMD: –0.02  (–0.037 to 0.003)*^,a^ | |  | | AMD: 0.002  (–0.021 to 0.025) | | | |  | |  |  | | |  |  |  |  |  |  | |
| **Taghizadeh et al 2019^34^**  N=953–1,237 (Total)  N=238–279 (+)   - Age: 50–75 years - Pan–Canadian Early Detection of Lung Cancer Study | Prospective cohort | LDCT | EQ-5D-3L | Total | 0.8 | | 0.8 | |  | |  | | | |  | |  |  | | |  |  |  |  |  | 0.8 | |
|  |  |  |  | (+) | 0.8 | | 0.8 | |  | |  | | | |  | |  |  | | |  |  |  |  |  | 0.8 | |
|  |  |  | EQ VAS | Total | 76.3 | | 76.8 | |  | |  | | | |  | |  |  | | |  |  |  |  |  | 76.8 | |
|  |  |  |  | (+) | 76.2 | | 76.1 | |  | |  | | | |  | |  |  | | |  |  |  |  |  | 76.4 | |
| **Ong et al 2016^50^**  N=234–327   - Age: ≥35+ years - HIV (+) MSM | Prospective questionnaire | DARE | SF-6D | Total (Abnormal, Normal) | 0.8 (0.1) | | 0.8 (0.1) | |  | | | | | | | |  |  | | |  |  |  |  |  |  | |
| **Korfage et al 2012^17^**  N=706–789   - Age: 30–60 years - Women living in Netherlands | Prospective cohort | Pap | EQ-5D | Normal | 0.9 (0.2) | | 0.9 (0.2)^b^ | | 0.9 (0.2)^c^ | | | |  | |  | |  |  | | |  |  |  |  |  |  | |
|  |  |  | EQ VAS |  | 81 (14) | | 81 (12)^b^ | | 82 (12)^c,^* | | | |  | |  | |  |  | | |  |  |  |  |  |  | |
| **van den Bergh et al 2011^48^**  N=143–684   - Age: 50–75 years - NELSON study participants | RCT | LDCT | EQ VAS | Indeterminate | 79.1 (77.8–80.5) | |  | |  | | 75.9 (73.4–78.4) | | | |  | |  |  | | |  |  |  |  |  |  | |
|  |  |  |  | (–) | 79.8 (78.6–81.1) | |  | |  | | 78.6 (76.2–81.0) | | | |  | |  |  | | |  |  |  |  |  |  | |
| **van den Bergh et al 2010^41^**  N=600–641   - Age: 50–75 years - NELSON study participants | RCT | LDCT | EQ VAS | Indeterminate | 79.1 (13.4) | | 78.3 (12.5)^d,^* | | 75.0 (14.5)* | | | |  | | | |  | 10.5%* | | |  | |  |  |  |  | |
|  |  |  |  | (–) | 79.4 (13.8) | | 79.4 (12.2)^d^ | | 79.2 (13.4) | | | |  | | | |  | NR | | |  | |  |  |  |  | |
| **van den Bergh et al 2008^43^**  N=288–324   - Age: 50–75 years - NELSON study participants | RCT | LDCT | EQ VAS | Total (–/Incidental) | 79.0^e^ | | 80.0^e^ | |  | | | |  | | | |  | 80.0^e,^* | | |  | |  |  |  |  | |

*Indicating statistical significance, *p*<0.05.

^†^SF-6D, EQ-5D: higher scores indicates better preference-weighted heath status; EQ VAS: higher scores indicated better self-rated health.

^a^Lower utility-based quality of life for those who perceived results as abnormal, compared to those who perceived their results as normal.

^b^Post-screening, with no specific time point.

^c^Post-screening, after receiving the results, with no specific time point.

^d^Post-screen, no results.

^e^Median values.

Abbreviations: AMD, adjusted mean difference; DARE, digital anal rectal examination; GBMSM, gay, bisexual, and other men who have sex with men; HRA, high resolution anoscopy; LDCT, low-dose computed tomography; MSM, men who have sex with men; NELSON, The Dutch-Belgian Randomized Lung Cancer Screening Trial; RCT, randomized controlled trial; SD, standard deviation; SF-6D, short-form 6 dimension; SPANC, Study of the Prevention of Anal Cancer; VAS, visual analog scale.
