# Supplementary material for: Patient-reported outcomes associated with cancer screening: a systematic review
Source: BMC Cancer. 2022 Mar 1;22:223. doi: 10.1186/s12885-022-09261-5 (PMC8886782; doi:10.1186/s12885-022-09261-5)
Supplement: Supplementary file 9 — Additional file 9: Table S9. Patient-Reported Outcome Related to Other Psychosocial. [file 12885_2022_9261_MOESM9_ESM.docx]

**Additional file 9: Table S9. Patient-Reported Outcomes Related to Other Psychosocial**

|  | **Study Design** | **Screening** | **Measure**^†^ | **Result** | |  | **Screening** | |  | **2 mo** | | **3 mo** | | **4 mo** | | **5 mo** | | **6 mo** | | **7 mo**–**–11 mo** | | | | | | **12 mo** | |
| --- | --- | --- | --- | --- | --- | --- | --- | --- | --- | --- | --- | --- | --- | --- | --- | --- | --- | --- | --- | --- | --- | --- | --- | --- | --- | --- | --- |
|  |  |  |  |  |  | **Baseline** | | **Within 1 mo** | |  |  |  |  |  |  |  |  |  |  | | | | | | | |  |
|  |  |  |  |  |  | **Mean (SD)** | | **Mean (SD)** | | **Mean (SD)** | | | | | | | | | | | | | | | | | |
| Cvejic et al 2020^67^  N=270–617   - Age: ≥35 years - HIV (+/–) GBMSM in the SPANC | Prospective cohort | Anal Swab + HRA | ASQ | Total (Abnormal, Normal) | More worried about getting cancer | NA | | NR*^,a^ | |  | | AMD: 0.8  (–2.7 to 1.1)^b^ | | |  | |  |  | | |  |  |  |  |  |  | |
|  |  |  |  |  | Felt they were more likely to develop cancer than other gay men their age |  |  | AMD: 0.3 (0.4–0.6)^a^ | |  | |  | | |  | |  |  | | |  |  |  |  |  |  | |
| Brain et al 2016^37^  N=1579–2019  N=73 (–)  N=41 (Incidental)  N=788 (+)/Repeat N=48 (+)/MDT   - Age: 50–75 years - UKLS trial participants | RCT | LDCT | SWD | Total (+/–/Incidental) | Not very satisfied | NA | | 58% | |  | |  | | |  | |  |  | | |  |  |  |  |  |  | |
|  |  |  |  |  | Very satisfied |  |  | 42% | |  | |  | | |  | |  |  | | |  |  |  |  |  |  | |
|  |  |  |  | (–) | Not very satisfied |  |  | 54% | |  | |  | | |  | |  |  | | |  |  |  |  |  |  | |
|  |  |  |  |  | Satisfied |  |  | 46% | |  | |  | | |  | |  |  | | |  |  |  |  |  |  | |
|  |  |  |  | Incidental | Not very satisfied |  |  | 56% | |  | |  | | |  | |  |  | | |  |  |  |  |  |  | |
|  |  |  |  |  | Satisfied |  |  | 44% | |  | |  | | |  | |  |  | | |  |  |  |  |  |  | |
|  |  |  |  | (+)/Repeat scan | Not very satisfied |  |  | 64% | |  | |  | | |  | |  |  | | |  |  |  |  |  |  | |
|  |  |  |  |  | Satisfied |  |  | 36%* | |  | |  | | |  | |  |  | | |  |  |  |  |  |  | |
|  |  |  |  | (+)/MDT referral | Not very satisfied |  |  | 43% | |  | |  | | |  | |  |  | | |  |  |  |  |  |  | |
|  |  |  |  |  | Satisfied |  |  | 57% | |  | |  | | |  | |  |  | | |  |  |  |  |  |  | |
|  |  |  |  | Control | Not very satisfied |  |  | 66% | |  | |  | | |  | |  |  | | |  |  |  |  |  |  | |
|  |  |  |  |  | Very satisfied |  |  | 34%* | |  | |  | | |  | |  |  | | |  |  |  |  |  |  | |
| Ong et al 2016^50^  N=234–327   - Age: ≥35+ years - HIV (+) MSM | Prospective questionnaire | DARE | NS | Total (Abnormal, Normal) | | NA | | During exam:   - Feeling in control (100%), relaxed (82%)   Experience after the exam:   - Optimistic about future health: 98% - Reassured by anal exam results: 83% - Life changed for the worse because of results: 2% | | | | | | | | |  |  | | |  |  |  |  |  |  | |
| Aggestrup et al 2012^40^  N=1,853– 2,052   - DLCST participants | RCT | LDCT | COS | Normal | Behavior | 0.7 (1.8) | |  | |  | |  | | |  | |  |  | | |  |  |  |  |  | 1.8 (2.9) | |
|  |  |  |  |  | Dejection | 1.2 (2.0) | |  | |  | |  | | |  | |  |  | | |  |  |  |  |  | 1.6 (2.7) | |
|  |  |  |  |  | Sleep | 0.6 (1.6) | |  | |  | |  | | |  | |  |  | | |  |  |  |  |  | 1.6 (2.5) | |
|  |  |  |  |  | Busy to take mind off things | 0.2 (0.6) | |  | |  | |  | | |  | |  |  | | |  |  |  |  |  | 0.3 (0.6) | |
|  |  |  |  |  | Less interest in sex | 0.5 (1.1) | |  | |  | |  | | |  | |  |  | | |  |  |  |  |  | 0.7 (1.3) | |
|  |  |  |  |  | Self-rated health | 1.0 (0.7) | |  | |  | |  | | |  | |  |  | | |  |  |  |  |  | 0.9 (0.8) | |
|  |  |  |  | Control | Behavior | 0.8 (2.1)* | |  | |  | |  | | |  | |  |  | | |  |  |  |  |  | 2.0 (3.0) | |
|  |  |  |  |  | Dejection | 1.4 (2.2)* | |  | |  | |  | | |  | |  |  | | |  |  |  |  |  | 1.9 (3.0)* | |
|  |  |  |  |  | Sleep | 0.7 (1.7) | |  | |  | |  | | |  | |  |  | | |  |  |  |  |  | 1.8 (2.6) | |
|  |  |  |  |  | Busy to take mind off things | 0.7 (1.7) | |  | |  | |  | | |  | |  |  | | |  |  |  |  |  | 0.3 (0.7) | |
|  |  |  |  |  | Less interest in sex | 0.7 (1.7) | |  | |  | |  | | |  | |  |  | | |  |  |  |  |  | 0.7 (1.3) | |
|  |  |  |  |  | Self-rated health | 1.0 (0.8) | |  | |  | |  | | |  | |  |  | | |  |  |  |  |  | 1.0 (0.8) | |
|  |  |  | COS-LC | Normal | Self-blame | NA | |  | |  | |  | | |  | |  |  | | |  |  |  |  |  | 1.2 (2.3) | |
|  |  |  |  |  | Focus on (airway) symptoms |  |  |  | |  | |  | | |  | |  |  | | |  |  |  |  |  | 3.3 (3.6) | |
|  |  |  |  |  | Stigmatization |  |  |  | |  | |  | | |  | |  |  | | |  |  |  |  |  | 1.2 (2.3) | |
|  |  |  |  |  | Introvert |  |  |  | |  | |  | | |  | |  |  | | |  |  |  |  |  | 1.9 (1.8) | |
|  |  |  |  |  | Harm of smoking |  |  |  | |  | |  | | |  | |  |  | | |  |  |  |  |  | 1.5 (1.7) | |
|  |  |  |  | Control | Self-blame |  |  |  | |  | |  | | |  | |  |  | | |  |  |  |  |  | 1.3 (2.4)* | |
|  |  |  |  |  | Focus on (airway) symptoms |  |  |  | |  | |  | | |  | |  |  | | |  |  |  |  |  | 3.8 (3.9)* | |
|  |  |  |  |  | Stigmatization |  |  |  | |  | |  | | |  | |  |  | | |  |  |  |  |  | 1.3 (2.4) | |
|  |  |  |  |  | Introvert |  |  |  | |  | |  | | |  | |  |  | | |  |  |  |  |  | 2.2 (3.0)* | |
|  |  |  |  |  | Harm of smoking |  |  |  | |  | |  | | |  | |  |  | | |  |  |  |  |  | 1.8 (2.9) | |
| Robb et al 2012^53^  N=674–913   - Age: 58–59 years - Registered at 34 London General Practices and part of NCS program | Prospective cohort | FS | Satisfaction with procedure | Total  (Low or high-risk polyps/–) | | NA | | NR | |  | |  | | | >95% | |  |  | | |  |  |  |  |  |  | |
|  |  |  | Colorectal symptoms |  |  | 2.0 (1.6)^c^ | |  | |  | |  | | | 1.2 (1.4)*^,c^ | |  |  | | |  |  |  |  |  |  | |

| Korfage et al 2012^17^  N=706–789   - Age: 30–60 years - Women living in Netherlands | Prospective cohort questionnaire | Pap | PCQ | Normal | | | | 0.8 (1.9) | 0.9 (2.0)^b^ | 0.8 (1.9)^d^ |  | |  |  |  |  |  |  |  |  |  |
| --- | --- | --- | --- | --- | --- | --- | --- | --- | --- | --- | --- | --- | --- | --- | --- | --- | --- | --- | --- | --- | --- |
|  |  |  | Satisfaction with procedure |  |  |  |  | 80% | NR^b^ | 66%^d^ |  | |  |  |  |  |  |  |  |  |  |
|  |  |  | Burden of procedure | Normal | | Very stressful | | 2% | NR^b^ | 1%^d^ |  | |  |  |  |  |  |  |  |  |  |
|  |  |  |  |  |  | Fairly stressful | | 23% | NR^b^ | 32%^d^ |  | |  |  |  |  |  |  |  |  |  |
|  |  |  |  |  |  | Not at all stressful | | 76% | NR^b^ | 67%^d^ |  | |  |  |  |  |  |  |  |  |  |
| Bredart 2012^75^  N=365–818   - Age: 20–70 years - Family history of breast cancer | Prospective observational | MMG | MGQ: Psychological experience | Normal | | | | NR | 23.4 (4.8) |  | |  |  |  |  |  |  |  |  |  |  |
|  |  | MRI+MMG |  |  |  |  |  |  | 24.6 (4.5)* |  | |  |  |  |  |  |  |  |  |  |  |
|  |  | MMG | MGQ: Perception of care |  |  |  |  | NR | 27.1 (3.6) |  | |  |  |  |  |  |  |  |  |  |  |
|  |  | MRI+MMG |  |  |  |  |  |  | 27.3 (3.7) |  | |  |  |  |  |  |  |  |  |  |  |
|  |  | MMG | Breast cancer risk perception |  |  |  |  | 0.4 (0.2)^e^  3.4% very high | 0.4 (0.2)^e^  3.3% very high |  | |  |  |  |  |  |  |  |  |  |  |
|  |  | MRI+MMG |  |  |  |  |  | 0.5 (0.2)^e^  14.9% very high | 0.5 (0.2)^e^  15.0% very high |  | |  |  |  |  |  |  |  |  |  |  |
|  |  | MMG | MRI-specific discomfort |  |  |  |  | NR | Duration: 35%  Immobility: 38%  Prone position: 21%  Noise: 65%  Panic feeling: 6%) |  | |  |  |  |  |  |  |  |  |  |  |
|  |  | MRI+MMG |  |  |  |  |  |  |  |  |  |  |  |  |  |  |  |  |  |  |  |
| **Byrne et al 2008^44^**  N=341   - Age: 50–79 years | Prospective cohort | LDCT | Perceived risk  of cancer | Suspicious | | | | 18.6 (15.7) | 34.5 (28.0)* |  | |  |  |  | 30.3 (28.0)* |  |  |  |  |  | 31.2 (28.9)* |
|  |  |  |  | Indeterminate | | | | 18.9 (22.9) | 20.1 (25.0) |  | |  |  |  | 14.8 (19.7) |  |  |  |  |  | 18.9 (25.2) |
|  |  |  |  | (–) | | | | 17.1 (20.4) | 11.2 (20.2) |  | |  |  |  | 13.1 (20.8) |  |  |  |  |  | 13.1 (19.9) |
| van den Bergh et al 2008^43^  N=322   - Age: 50–75 years - NELSON study participants | RCT | LDCT | Discomfort questionnaire | Total  (–/ incidental) | | Discomfort from waiting for  CT scan results | | NA | 76.0% |  | |  |  |  |  |  |  |  |  |  |  |
|  |  |  |  |  |  | Prospect of CT scanning | |  | 17.8% |  | |  |  |  |  |  |  |  |  |  |  |
|  |  |  |  |  |  | Undergoing CT scanning | |  | 6.2% |  | |  |  |  |  |  |  |  |  |  |  |
| Bunge et al 2008^49^  N=40–47 (High AR)  N=236–274  (Low AR)   - Age: 50–75 years - NELSON study participants | RCT | LDCT | Perceived risk of cancer | High AR | | | | 14.5% |  |  | |  | |  | 10.5%* |  | |  |  |  |  |
|  |  |  |  | Low AR | | | | NR |  |  | |  | |  | NR |  | |  |  |  |  |
| Tyndel et al 2007^52^  Recall: 120–145  Clear: 1316–1448   - Age: 35–49 years - Women with a moderate or high risk of familial breast cancer | Prospective cohort | MMG | PCQ | Recall | | | | 7.3 (7.7) | 7.1 (7.4) |  | |  | |  | 4.6 (6.4) |  | |  |  |  |  |
|  |  |  |  | All-clear | | | | 5.1 (6.7) | 4.2 (6.2) |  | |  | |  | 3.8 (6.0) |  | |  |  |  |  |
| Taylor et al 2004^61^  N=163–325   - Age: 55–74 years - PLCO cancer screening trial participants - Family history or FDR with cancer | Randomized screening | DRE + PSA; CXR; FS;  TVS + CA125 | SWD | Screen | | | | 4.4 (0.4) | NR |  | |  | |  |  |  | |  |  |  | NR |
|  |  |  |  | Normal | | | |  | 4.6 (0.5) |  | |  | |  |  |  | |  |  |  | 4.5 (0.5) |
|  |  |  |  | Abnormal | | | |  | 4.6 (0.5) |  | |  | |  |  |  | |  |  |  | 4.5 (0.5) |
|  |  |  |  | Control | | | | 4.5 (0.5) | NR |  | |  | |  |  |  | |  |  |  | 4.3 (0.6) |
| Absetz et al 2003^45^  N=145–643   - Age: 50–59 years - Some participants with familial history of breast cancer | Prospective questionnaire | MMG | Perceived susceptibility (perceived personal lifetime risk) | Normal | High PS | | Low risk | 5.4% |  |  | |  | |  |  |  | |  |  |  | 12.0% |
|  |  |  |  |  |  |  | Mod risk | 56.2% |  |  | |  | |  |  |  | |  |  |  | 49.0% |
|  |  |  |  |  |  |  | High risk | 17.7% |  |  | |  | |  |  |  | |  |  |  | 12.0% |
|  |  |  |  |  | Mod PS | | Low risk | 21.5% |  |  | |  | |  |  |  | |  |  |  | 27.4% |
|  |  |  |  |  |  |  | Mod risk | 42.6% |  |  | |  | |  |  |  | |  |  |  | 38.4% |
|  |  |  |  |  |  |  | High risk | 6.4% |  |  | |  | |  |  |  | |  |  |  | 4.0% |
|  |  |  |  |  | Low PS | | Low risk | 45.5% |  |  | |  | |  |  |  | |  |  |  | 45.6% |
|  |  |  |  |  |  |  | Mod risk | 28.0% |  |  | |  | |  |  |  | |  |  |  | 22.3% |
|  |  |  |  |  |  |  | High risk | 2.3% |  |  | |  | |  |  |  | |  |  |  | 1.0% |
|  |  |  |  | FP | High PS | | Low risk | 7.0% |  |  | |  | |  |  |  | |  |  |  | 7.0% |
|  |  |  |  |  |  |  | Mod risk | 47.0% |  |  | |  | |  |  |  | |  |  |  | 43.0% |
|  |  |  |  |  |  |  | High risk | 23.0% |  |  | |  | |  |  |  | |  |  |  | 27.0% |
|  |  |  |  |  | Mod PS | | Low risk | 17.0% |  |  | |  | |  |  |  | |  |  |  | 23.6% |
|  |  |  |  |  |  |  | Mod risk | 38.0% |  |  | |  | |  |  |  | |  |  |  | 44.3% |
|  |  |  |  |  |  |  | High risk | 9.0% |  |  | |  | |  |  |  | |  |  |  | 11.4% |
|  |  |  |  |  | Low PS | | Low risk | 42.0% |  |  | |  | |  |  |  | |  |  |  | 51.0% |
|  |  |  |  |  |  |  | Mod risk | 27.0% |  |  | |  | |  |  |  | |  |  |  | 33.0% |
|  |  |  |  |  |  |  | High risk | 4.0% |  |  | |  | |  |  |  | |  |  |  | 5.0% |

^†^PCQ: higher scores indicating greater psychological consequences of screening; SWD: higher scores indicating greater satisfaction with the decision to participate in the trial; COS: higher scores indicated more negative psychosocial consequences; Affective risk perception: higher numbers indicating higher perceived risk of cancer; MGQ: higher scores indicate a positive judgment of psychological experience and perception.

^a^A greater proportion of participants who perceived their results as abnormal reported feeling more worried about getting anal cancer at both time points (both *p*<0.05) and felt more likely to develop anal cancer than other gay men their age at both time points (both *p*<0.05), compared with those who perceived their results as normal.

^b^Post screen, with no specific time point.

^c^Indicates average number of symptoms.

^d^After receiving screen results, with no specific time point.

^e^A total score may be computed ranging between 0 (very low perceived risk) and 1 (very high perceived risk).

Abbreviations: AMD, adjusted mean difference; AR, affective risk; ASQ, anal screening questionnaire; CA-125, ovarian tumor marker; COS-LC; Consequences Of Screening in Lung Cancer; CT, computed tomography; CXR, chest x-ray; DARE, digital anal rectal examination; DRE, digital rectal exam; DLCST, Danish Lung Cancer Screening Trial; FDR, first degree relative; FP, false positive; FS, flexible sigmoidoscopy; GBMSM, gay, bisexual, and other men who have sex with men; HRA, high resolution anoscopy; LDCT, low-dose computed tomography; MDT, multidisciplinary team; MGQ, MammoGraphy Questionnaire; MMG, mammogram; MRI, magnetic resonance imaging; MSM, men who have sex with men; NA, not applicable; NCS, national cancer screening; NR, not reported; NS, not specified; PCQ, Psychological Consequences Questionnaire; PLCO, prostate, lung, colorectal, and ovarian; PS, perceived susceptibility; PSA, prostate-specific antigen; RCT, randomized controlled trial; SD, standard deviation; SPANC, Study of the Prevention of Anal Cancer; SWD, satisfaction with decision to participate in the trial; TVS, transvaginal ultrasound scan; UKLS, United Kingdom Lung Screening.
